# Detection of novel syntrophic acetate‐oxidizing bacteria from biogas processes by continuous acetate enrichment approaches

**DOI:** 10.1111/1751-7915.13035

**Published:** 2017-12-04

**Authors:** Maria Westerholm, Bettina Müller, Abhijeet Singh, Oskar Karlsson Lindsjö, Anna Schnürer

**Affiliations:** ^1^ Uppsala BioCenter Department of Molecular Sciences Swedish University of Agricultural Sciences Box 7025 SE‐750 07 Uppsala Sweden

## Abstract

To enrich syntrophic acetate‐oxidizing bacteria (SAOB), duplicate chemostats were inoculated with sludge from syntrophic acetate oxidation (SAO)‐dominated systems and continuously supplied with acetate (0.4 or 7.5 g l^−1^) at high‐ammonia levels. The chemostats were operated under mesophilic (37°C) or thermophilic (52°C) temperature for about six hydraulic retention times (HRT 28 days) and were sampled over time. Irrespective of temperature, a methane content of 64–69% and effluent acetate level of 0.4–1.0 g l^−1^ were recorded in chemostats fed high acetate. Low methane production in the low‐acetate chemostats indicated that the substrate supply was below the threshold for methanization of acetate via SAO. Novel representatives within the family Clostridiales and genus *Syntrophaceticus* (class Clostridia) were identified to represent putative SAOB candidates in mesophilic and thermophilic conditions respectively. Known SAOB persisted at low relative abundance in all chemostats. The hydrogenotrophic methanogens *Methanoculleus bourgensis* (mesophilic) and *Methanothermobacter thermautotrophicus* (thermophilic) dominated archaeal communities in the high‐acetate chemostats. In line with the restricted methane production in the low‐acetate chemostats, methanogens persisted at considerably lower abundance in these chemostats. These findings strongly indicate involvement in SAO and tolerance to high ammonia levels of the species identified here, and have implications for understanding community function in stressed anaerobic processes.

## Introduction

Syntrophic acetate‐oxidizing bacteria (SAOB) drive anaerobic conversion of acetate to methane in high‐ammonia biogas processes and thus play a major role in many commercial‐scale production systems (Karakashev *et al*., 2006; Sun *et al*., 2014; Frank *et al*., 2016; Mosbaek *et al*., 2016). SAOB work in close association with hydrogenotrophic methanogens, performing a two‐step reaction in which SAOB convert acetate to H_2_/format and CO_2_, which are then used by the methanogens for production of CH_4_ and CO_2_. Under conditions such as high ammonia, the syntrophic acetate oxidizers outnumber their competitors for acetate, the aceticlastic methanogens (Sun *et al*., 2014). This poses challenges regarding biogas digester operation, as low‐acetate conversion rates by the syntrophs may limit the overall efficiency and stability of the process. However, tailoring operation to underpin the syntrophic interactions, such as allowance of microbial adaptation during start‐up and changed operating conditions, long retention times and addition of trace elements has been shown to be effective in improving performance and mitigates the effect of ammonia toxicity in SAO processes (Westerholm *et al*., 2016).

To date, only a few SAOB have been isolated and characterized. These are the thermophilic *Thermacetogenium phaeum* (Hattori *et al*., 2000) and *Pseudothermotoga lettingae* (Balk *et al*., 2002; Bhandari and Gupta, 2014), the thermotolerant *Tepidanaerobacter acetatoxydans* (Westerholm *et al*., 2011a,b) and the mesophilic [*Clostridium*] *ultunense* (Schnürer *et al*., 1996) and *Syntrophaceticus schinkii* (Westerholm *et al*., 2010). Presence and abundance of these SAOB during changed operating conditions in anaerobic systems have been indicated using species‐specific 16S rRNA gene‐targeting approaches (Westerholm *et al*., 2011a,b, 2012a,b, 2015). However, known SAOB are often low in abundance in relation to the overall microbial community (Westerholm *et al*., 2016). Their relative low abundance obstructs their detection, but also imposes limitations for identification of new potential SAOB using high‐throughput sequencing of complex anaerobic digester communities. Nevertheless, altering process operation towards SAO‐inducing conditions (such as increased ammonia level, injection of H_2_ or elevated temperature) may affect the microbial community enough for detection of potential SAOB using sequencing approaches. Through such approaches, SAOB candidates have been suggested within the families Thermoanaerobacteraceae (which also includes the known SAOB *T. phaeum* and *S. schinkii*) and Thermodesulfobiaceae (Ho *et al*., 2014; Yamada *et al*., 2014; Bassani *et al*., 2015; Müller *et al*., 2016) and the phylum Spirochaetes (Lee *et al*., 2015). However, the inconceivable numbers of microbes and high microbial diversity in anaerobic digesters pose a major challenge when seeking to establish reliable links between abundant species and SAO‐function and further research is required to confirm species within these groups as SAOB.

Most known SAOB are affiliated to the physiological group of acetogens, a feature that has been used to reveal further information about potential SAOB by targeting the *fhs* gene, encoding a key enzyme of both acetogenic and SAO metabolism (Müller *et al*., 2016). Through this method, acetogenic groups unique to high‐ammonia biogas processes have been identified and are suggested to be involved in SAO (Müller *et al*., 2016). Results of other techniques to track down potential SAOB, such as stable isotope‐based functional probing and meta‐omics, suggest members of the orders Clostridiales and/or Thermoanaerobacterales (Zakrzewski *et al*., 2012; Lü *et al*., 2014; Müller *et al*., 2016), uncultured phylotypes affiliated with the Firmicutes (Frank *et al*., 2016), the Thermotogae (Zakrzewski *et al*., 2012; Nubo *et al*., 2015) and the phylum Synergistes (Ito *et al*., 2011) as candidates for SAO capacity. Taken together, the few SAOB isolates and the wide taxonomic diversity of the proposed SAOB currently pose an obstacle to predicting their function and behaviours in complex microbial communities. Identification of novel key players would thus be highly beneficial and would increase knowledge of SAO and help manage ammonia‐stressed anaerobic digesters and develop innovative operating guidance.

The aim of this study was to enrich acetate‐degrading microbial communities using a continuous cultivation approach to preserve and enrich core acetate‐utilizing communities occurring in high‐ammonia biogas systems. Continuous feeding with acetate for a long period was expected to enable microbial enrichment and facilitate identification of prominent SAOB, which due to their relatively low abundance are difficult to detect in more complex environments. The enrichments were initiated with inocula taken from anaerobic digesters previously demonstrated to be dominated by SAO. Differing factors between the enrichment chemostats included acetate influent concentration (0.4 or 7.5 g l^−1^), temperature (37°C or 52°C) and inoculum source, which were selected with the objective of enriching SAO populations, occupying different niches with regard to acetate concentration and optimal temperature conditions. Other operating parameters of the parallel acetate enrichments were set to mimic the continuous biogas system that was the source of the inoculum, that is high free ammonia level (0.6–0.9 g NH_3_ l^−1^) and ~30 day retention time. A combination of molecular methods, including Illumina sequencing of 16S rRNA genes, quantitative polymerase chain reaction (qPCR) and terminal restriction fragment length polymorphism (T‐RFLP) analysis, was used to identify microbial structure patterns over time and to quantify abundant species. This approach made it possible to focus on the metabolic group restricted to acetate degradation.

## Results

### Anaerobic chemostat performance

The mesophilic high‐acetate (7.5 g l^−1^) chemostats (M_H_) had a mean acetate effluent of 0.5 ± 0.1 g l^−1^ during hydraulic retention times (HRT) 2–6. During the corresponding period, the thermophilic high‐acetate chemostats (T_H_) had significantly higher values, averaging 1.2 ± 0.2 g acetate l^−1^ in T_H1_ and 0.7 ± 0.2 g acetate l^−1^ in T_H2_ (Fig. S1, Table S1). The high‐acetate chemostats produced gas with high methane content around 64–69% both at mesophilic and thermophilic conditions. All low‐acetate (0.4 g l^−1^) chemostats (M_L_, T_L_) had mean acetate effluent below the detection limit of 0.1 g l^−1^ and the methane level was as low as 0.3–9% throughout the trial. Ammonia concentration (g NH_3_ l^−1^) varied between 0.3–0.4 in M_L_, 0.7–0.8 in M_H_, 0.8–1.0 in T_L_ and 1.5–1.8 in T_H_ due to the different pH and temperature in the chemostats (Table S1).

As expected, propionate, butyrate, isobutyrate, valerate, isovalerate, capronate and isocapronate were not detected in any of the chemostats. Irrespective of operating temperature, pH remained stable over time around 7.7–7.8 and 8.1–8.2 in low‐acetate and high‐acetate chemostats respectively (Table S1).

### Illumina sequencing and diversity indices

Illumina sequencing identified a total of 36 395 830 reads, of which 10 240 118 were of high quality. Rarefaction analysis is displayed in Fig. S2. The estimated coverage indicated that on average 79 ± 1% of the microbial community was covered in all samples. Alpha diversity of microbial communities showed significantly decreased richness and evenness (S_obs_, Chao1 and Shannon diversity) throughout operation in all mesophilic chemostats and in the T_H_ chemostats, whereas in T_L_ there was no difference in alpha diversity values over time (Fig. S3, Tables S2 and S3). Richness and evenness indices in the high‐acetate chemostats were significantly higher in mesophilic than in thermophilic chemostats (Table S4).

### Bacterial community structure and dynamics in mesophilic chemostats

Community OTU comparison by non‐metric multidimensional scaling (NMDS; OTU ≥ 97% identity) of each sample using the Bray–Curtis similarity metric revealed close grouping of M_H_ chemostats, whereas the microbial communities in the low‐acetate chemostats drifted apart over time (Fig. S4A).

**Figure 1:**
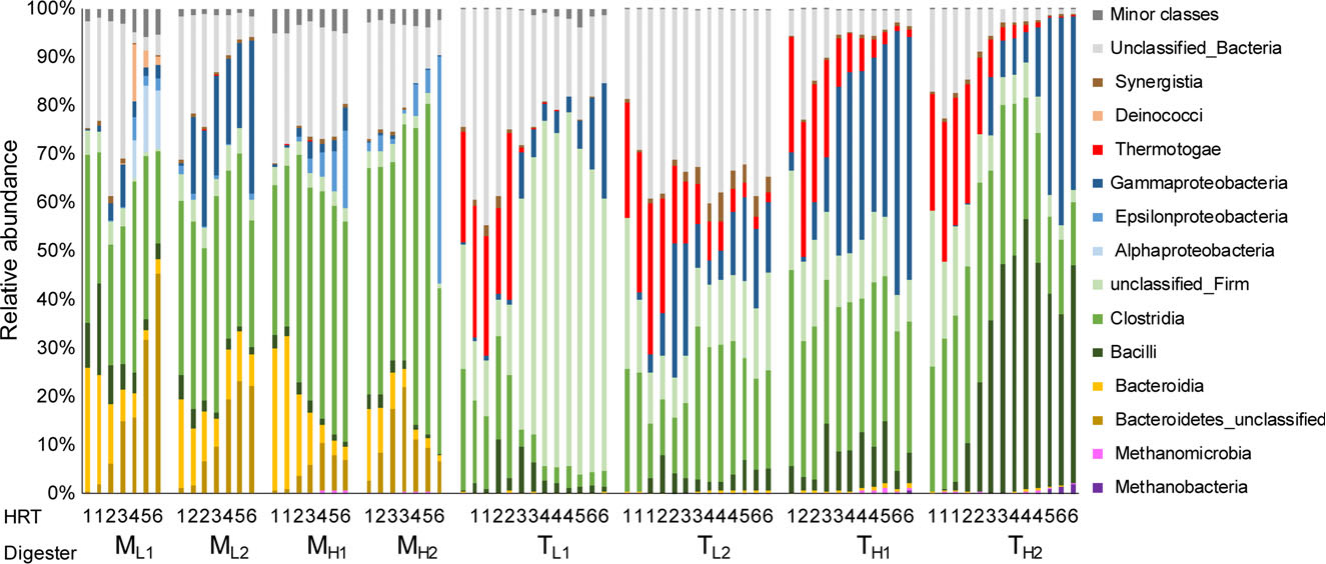
Percentage contribution of 16S rRNA gene sequences (grouped by class) during acetate enrichment under mesophilic (M) or thermophilic (T) temperature conditions, as determined using Illumina MiSeq sequencing. Chemostats designated M_H_/T_H_ and M_L_/T_L_ received medium containing 7.5 and 0.4 g acetate l^−1^ respectively. The corresponding hydraulic retention time (HRT) at the point of sampling is given on the *x*‐axis. Yellow, green, blue and red bars represent classes within the phyla Bacteriodetes, Firmicutes, Proteobacteria and Thermotogae respectively. Purple indicates methanogenic archaea. Classes representing < 1% were included in minor classes.

Firmicutes (37–53%) and Bacteroidetes (17–30%) were the most abundant phyla during initial operation of the four mesophilic chemostats (Fig. 1). The majority of the remaining bacterial sequences were not classified at phylum level (22–30% of total reads). In both M_H_ chemostats, Firmicutes remained abundant at 35–71% of total sequences throughout operation, whereas Bacteroidetes gradually decreased to constitute 8–13% after 4 HRT. Proteobacteria became more abundant over time and represented 22–47% after 4 HRT (initial level ≤ 2%). Microbial dynamics in M_L_ chemostats included increased relative abundances of Proteobacteria (to 18–22%) and Bacteroidetes (to 29–49%) and stable proportions of Firmicutes (23–42% after 4 HRT), whereas unclassified bacteria decreased to < 8% (Fig. 1).

On a class taxonomic scale, Clostridia (phylum Firmicutes) dominated the microbial communities in M_H_ and was foremost comprised of one OTU (designated OTU_M_H_C), belonging to the family Clostridiaceae and sharing 96% identity with *Alkaliphilus oremlandii* and *Alkaliphilus halophilus*. The relative abundance of OTU_M_H_C increased from 2% to 3% during initial operation to 12–23% in M_H1_ and 26–44% in M_H2_ at the end of the experimental period. In M_L_, the relative abundance of OTU_M_H_C remained below 3%.

Epsilonproteobacteria was the dominant class in Proteobacteria in M_H_ chemostats, and the vast majority of sequences were from an OTU associated with the genus *Sulfurospirillum* (96% gene sequence identity to *Sulfurospirillum alkalitolerans*). During operation of the M_H_ chemostats, this *Sulfurospirillum* sp. increased from < 3% in initial operation to 5–8% after 4 HRT and 16–47% in final HRT. Under lower acetate conditions, other classes dominated the phylum Proteobacteria, that is Alphaproteobacteria in M_L1_ and Gammaproteobacteria in M_L2_. In the class Gammaproteobacteria, the most dominant populations belonged to the family Pseudomonadaceae. These Pseudomonadaceae sp. were also found in thermophilic enrichment chemostats.

**Figure 2:**
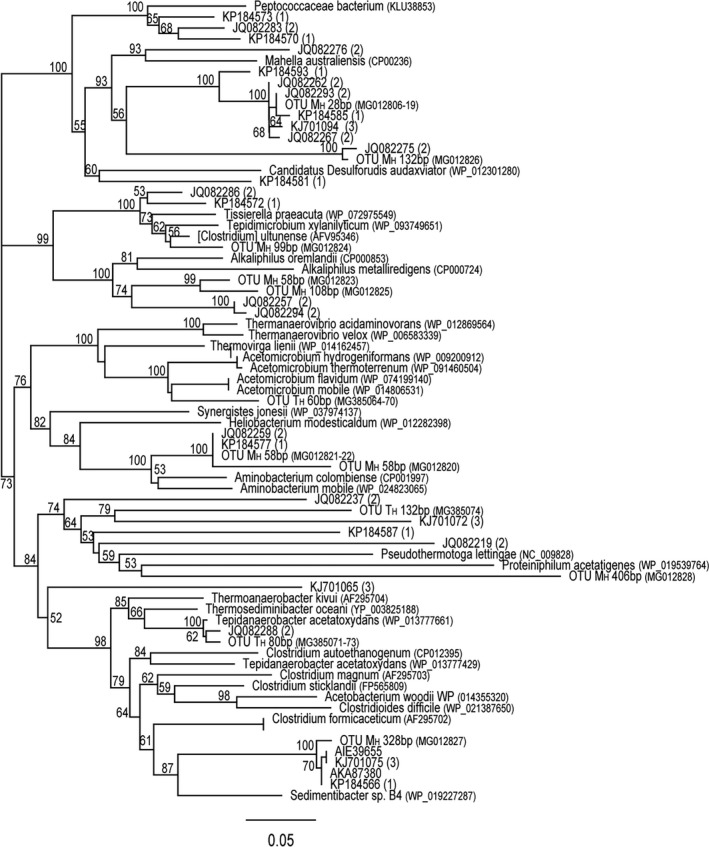
Phylogenetic placement of deduced FTHFS amino acid sequences of the partial *fhs* sequences retrieved from the high‐acetate mesophilic (M_H_) and thermophilic (T_H_) chemostats. Accession numbers are given for reference strains and for *fhs* gene sequences retrieved in this study (OTU M_H_/T_H_) and in previous studies of SAO‐dominated anaerobic digesters: (1) (Moestedt *et al*., 2016), (2) (Müller *et al*., 2016), (3) (Westerholm *et al*., 2015). The SAOB *S. schinkii* and *T. phaeum* and sulfate‐reducing bacteria formed a separate clade and were excluded from the alignment to reduce the size of the tree. The scale bar represents 50% sequence divergence. Values at the nodes are the percentage of 1000 bootstrap replicates; values below 50% are not shown.

T‐RFLP analysis was performed to characterize the dynamics of the SAOB community by targeting the formyltetrahydrofolate synthetase (*fhs*) gene, a functional marker for SAOB and the acetogenic community in general. The majority of the fragments appearing in the high‐acetate chemostats were phylogenetically assigned by means of clone library analyses (yield of 35 partial *fhs* gene sequences in total). The T‐RFLP analysis showed some discrepancies between samples taken from initial operation of the duplicate M_H_ chemostats (Fig. S5A). However, both replicate communities had a high relative abundance of a restriction fragment of 28 bp, representing 15–74% in the M_H_ chemostats. A 58 bp restriction fragment constituted 6–27% in M_H1_ throughout operation, whereas in M_H2_ this fragment decreased from 8–17% to below detection limit at HRT 5–6. A fragment of 108 bp was detected in abundance of 4–15% at HRT 4–6 (except in M_H1_ at HRT 5). Throughout operation of M_H_, the T‐RFs 328 and 406 bp significantly decreased and increased respectively (Fig. S5A, Table S2). None of the T‐RFs that were highly abundant in later operation of M_H_ were detected in the M_L_ communities after 6 HRT. Clone sequences likely representing restriction fragments of 28, 58, 108 and 328 bp in the T‐RFLP had low nucleotide sequence identity (< 79%) to previously characterized species, but showed 97–99% identity to *fhs* gene sequences previously retrieved from high‐ammonia digesters (Westerholm *et al*., 2015; Moestedt *et al*., 2016; Müller *et al*., 2016) (Table S5). The T‐RF 406 bp had low sequence identity (< 79%) to previously recovered *fhs* genes but positioned close to the SAOB *P. lettingae* in the phylogenetic analyses (Fig. 2, Table S5).

**Figure 3:**
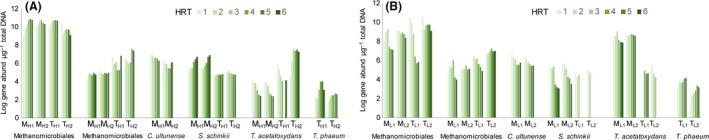
Log gene abundances of the methanogens Methanomicrobiales and Methanobacteriales and the syntrophic acetate‐oxidising bacteria (SAOB) *C. ultunense*,* S. schinkii*,* T. acetatoxydans* and *T. phaeum* in duplicate mesophilic (M) and thermophilic (T) chemostat fed 7.5 (A, _H_) and 0.4 (B, _L_) g acetate l^−1^. Each hydraulic retention time (HRT) represents 28 days of operation.

Quantitative PCR analyses of known SAOB revealed stable abundance of *C. ultunense* (2.3 ± 0.5 × 10^6^ gene copies μg^−1^ DNA) in all mesophilic chemostats, irrespective of acetate level. Over time, *S. schinkii* significantly increased in M_H_ (from 10^5^ to 10^7^ gene copies μg^−1^ DNA) and decreased in M_L_ chemostats (from 10^5^ to 10^3–4^ gene copies μg^−1^ DNA) and was thus significantly higher under high‐acetate than low‐acetate conditions after 2 HRT (Fig.ucode> 3, Table S2). *T*. *acetatoxydans* decreased in all mesophilic chemostats and was even below the limit of detection in M_L1_ after 2 HRT (Fig. 3, Table S2). *T*. *phaeum* was not detected in any mesophilic chemostat. *Fhs* gene abundances of potential SAOBs (previously identified in high‐ammonia processes; Müller *et al*., 2016) were stable at around 10^6^ for *fhs*OTU8‐9 and at twofold higher values for *fhs*OTU3‐5 throughout operation of the M_H_ chemostats. In the M_L_ chemostats, these OTUs continually declined and became less abundant than in M_H_ after 2 HRT. *fhs*OTU10 significantly decreased in all mesophilic chemostats (Fig. S6, Table S2). The total bacteria determined by qPCR analyses showed stable levels in all mesophilic chemostats relative to total extracted DNA (averaged 5.1 ± 0.5 × 10^8^ gene copies μg^−1^ DNA). However, when analysed per volume chemostat sludge, the total bacteria decreased over time in M_L_ (from 10^8^ to 10^7^ gene copies ml^−1^), whereas the levels remained relatively stable at around 10^8^ gene copies ml^−1^ in the M_H_ chemostats (Fig. S7A).

### Methanogenic community structure and dynamics in mesophilic chemostats

Illumina sequencing revealed < 0.02% methanogenic reads during initial operation of the M_H_ chemostats and throughout complete operation of the M_L_ chemostats. The relative contribution of methanogens increased to 0.1–3.0% over time in M_H_ (HRT 2‐6), primarily due to increased relative abundance of an OTU with 100% 16S rRNA gene sequence identity with *Methanoculleus bourgensis*. The mcrA (methyl coenzyme‐M reductase) genes retrieved from the duplicate M_H_ chemostats after 6 HRT showed 100% identity to a mcrA gene previously recovered in the biogas digester that was the source of the inoculum for the enrichment chemostats (OTU15 in D^TE^37, accession number KJ701175; Westerholm *et al*., 2015). The qPCR analyses revealed significantly (*P *<* *0.001) higher Methanomicrobiales abundance in M_H_ (3.7 ± 0.7 × 10^10^ gene copies μg^−1^ DNA) than in M_L_ (7.2 ± 2.0 × 10^8^ gene copies μg^−1^ DNA) at HRT 2‐6 (Fig. 3, Table S2). In contrast, the differences in abundance of Methanobacteriales in high‐acetate (6.6 ± 0.7 × 10^4^ gene copies μg^−1^ DNA) compared with low‐acetate chemostats (1.9 ± 0.6 × 10^5^ gene copies μg^−1^ DNA) were not statistically (*P *>* *0.05) significant. Methanosarcina were not detected in any chemostat.

### Bacterial community structure and dynamics in thermophilic chemostats

Non‐metric multidimensional scaling visualization showed separated clustering by chemostats. The microbial community structure in all chemostats was highly dynamic over time, in particular in T_L1_ (Fig. S4B). The phyla Firmicutes (47–74%) and Thermotogae (16–29%) were the most abundant bacterial populations in the first and second HRT under thermophilic conditions at both acetate levels (Fig. 1). Thermotogae thereafter decreased significantly in all thermophilic chemostats and represented < 8% at HRT 4–6. Firmicutes remained relatively constant, whereas the relative representation of Proteobacteria increased from < 4% to 3–24% in T_L_, 32–54% in T_H1_ and 6–43% in T_H2_ after HRT 4. In T_H_, unclassified bacteria comprised < 3% during the final operating period, whereas in T_L_ about 14–39% of the total reads were not classified at phylum level (Fig. 1).

Although the relative abundance of Firmicutes remained at a similar level throughout operation, the relative contribution of the family Thermoanaerobacteraceae within this phylum rapidly increased, from 2–3% at HRT 1 to 8–27% at HRT 2–6, in the T_H_ chemostats. The majority of the Thermoanaerobacteraceae sequences clustered into one OTU (referred to as OTU_T_H_S), with a representative sequence that exhibited 97% and 93% identity to the previously identified mesophilic SAOB *S. schinkii* and thermophilic SAOB *T. phaeum* respectively. In T_L_, the relative abundance of OTU_T_H_S accounted for < 2% of total sequences at all sampling points. The genus *Tepidimicrobium* (phylum Firmicutes, class Clostridia) was initially present at 7–22% in all thermophilic samples, but decreased below detection after a few retention times. An OTU, of which the representative sequence showed an identical match to the previously characterized bacteria *Bacillus infernus* (phylum Firmicutes, class Bacillus), increased in relative abundance over time in T_H2_ and represented 34–53% between HRT 3–6. The relative abundance of this OTU was < 10% in *T*_H1_ and *T*_L_ chemostats. Large proportions of the phylum Firmicutes were unclassified below phylum level in *T*_L1_, whereas in *T*_L2_ the majority of the sequences belonged to the class Clostridia (Fig. 1).

Gammaproteobacteria was the dominant class in Proteobacteria, mainly constituted by two OTUs classified into the family Pseudomonadaceae. These OTUs were closely related (96.8% gene sequence similarity) and together represented 3–22% and 6–53% at HRT 3‐6 in T_L_ and T_H_ chemostats respectively. These OTUs were also found to be highly abundant (17–32% of total sequences) in later operation of the low‐acetate mesophilic M_L2_ chemostat.

T‐RFLP analysis of the T_H_ chemostats identified a restriction fragment of 536 bp that significantly increased from 10–11% to 32–35% during the course of the experiment (Fig. S5B). T‐RFs 60, 132 and 636 bp were other highly abundant restriction fragments in one or both duplicate chemostats during the operating trial. However, the majority of the abundant T‐RFs in the T_H_ chemostats also represented 9–29% of the total *fhs* genes recovered in T_L2_ at HRT 6, which obstructed interpretation of their correlation to acetate degradation. Sequencing of *fhs* genes allowed phylogenetic assignment of T‐RFs 60, 80 and 132 bp in the T‐RFLP (in total 12 clones, Table S5). T‐RF 80 bp had close identity to *T. acetatoxydans* (96%) and *fhs* genes previously retrieved from a high‐ammonia SAO‐dominated digester (Müller *et al*., 2016). The 536 and 636 bp fragments appearing at high abundance in the T‐RFLP were not matched in the clone library. Furthermore, none of the *fhs* genes was closely related to *S. schinkii* and *T. phaeum* FTHFS, and they were thus not included as reference strains in the phylogenetic analyses (Fig. 2).

The 16S rRNA gene‐targeting qPCR analyses revealed constant levels of *S. schinkii* in T_H_ (4.0–8.7 × 10^4^ gene copies μg^−1^ DNA), whereas this species was not detected in T_L_ after 3 HRT. *T. phaeum* increased from 10^1^ to 10^3^ gene copies μg^−1^ DNA during the experimental trial in all chemostats. *T. acetatoxydans* decreased to below detection in T_H1_, but in T_H2_, it increased from 10^5–6^ to 10^7–8^ gene copies μg^−1^ DNA after 3 HRT (Fig. 3). *C. ultunense* 16S rRNA genes and *fhs* genes previously linked to potential SAOB (Müller *et al*., 2016) were not detected in any of the thermophilic chemostats.

Total bacterial abundances per ml sludge declined significantly in T_H_ chemostats and T_L2_ (Fig. S7B, Table S3). However, in T_L1_, the levels were qualitatively similar throughout operation (Fig. S7B), although, as in the mesophilic chemostats, the relative abundance observed over time showed similar levels in all chemostats when related to total DNA. This supports the results obtained in comparative analyses of the chemostats.

### Methanogenic community structure and dynamics in thermophilic chemostats

Methanogenic relative abundances did not exceed 0.3% in the Illumina sequencing data for the T_L_ chemostats. In the T_H_ chemostats, the methanogenic contribution increased from < 0.1% to 5–10% of total sequences by the end of the trial. The Illumina sequencing indicated equal relative abundance of an OTU belonging to the genus *Methanothermobacter* (order Methanobacteriales, 100% similarity to *M. thermophilus*,* M. wolfeii* and *M. defluvii*) and an OTU with identical sequence to *M. bourgensis* (type strain MS_2_T, order Methanomicrobiales) in T_H1_. In T_H2_, Methanothermobacter was the dominant methanogen according to the Illumina sequencing. However, based on qPCR data, Methanobacteriales abundance was 3.4 ± 1.8 × 10^6^ in T_H1_ and 3.3 ± 1.0 × 10^7^ in T_H2_ at HRT 5–6, which was several orders of magnitude lower than Methanomicrobiales abundance (4.5 ± 0.8 × 10^10^ gene copies μg DNA^−1^ in both T_H_ chemostats; Fig. 3, Table S3).

## Discussion

### Possible acetate dependencies of SAOB yet to be revealed

A continuous enrichment approach was employed in the present study to select for syntrophic acetate oxidizers, which due to their relatively low abundance are difficult to detect in more complex environments. The experimental set‐up was designed to distinguish mesophilic and thermophilic key populations during continuous feeding of two different levels of acetate. Acetate‐dependent growth rate of SAOB has been indicated in co‐cultivation and metagenomic studies (Oehler *et al*., 2012; Manzoor *et al*., 2015; Müller *et al*., 2015; Westerholm *et al*., 2016). Estimation of the degree to which the acetate level shapes syntrophic acetate‐degrading communities in biogas digesters is complex, particularly as high acetate levels often co‐occur with high levels of ammonia, which is a strong driver of development of the microbial community (Werner *et al*., 2014; De Vrieze *et al*., 2015; Müller *et al*., 2016). The set‐up of the present study was designed to shed more light on this subject. However, absence of methane formation in the mesophilic and thermophilic low‐acetate chemostats indicated that the influent acetate level was below the threshold for methane production via SAO. Aceticlastic methanogens are known to degrade acetate to concentrations below that level (Smith and Ingram‐Smith, 2007); however, in present chemostats, they were likely inhibited by the high ammonia level. Nevertheless, acetate was apparently consumed, judging by the lower acetate level in effluent than in influent. Consistent with this, Illumina sequencing analyses indicated presence of relatively diverse bacterial communities in M_L_ and T_L_. These microorganisms might degrade acetate and/or remain in the chemostats by consuming compounds included in the medium for growth support or as reducing agents (i.e. yeast extract, cysteine). However, the microbial community profiles obtained for the low‐acetate chemostats were still useful as references to distinguish SAOB candidates prevalent in M_H_ and T_H_. Given the declining trends in *S. schinkii* and *T. acetatoxydans* abundance in the M_L_ and T_L_ chemostats, the acetate level in the chemostats was below the threshold for SAO activity of these known SAOB. However, the acetate level did not affect the abundance of *C. ultunense*, indicating that this species was able to stay syntrophically active at low acetate levels or used cysteine supplied in the medium for its growth (Schnürer *et al*., 1996).

In particular at thermophilic temperature, the overall community sequencing and the T‐RFLP profiling revealed surprisingly high structural variations between duplicate chemostats (representing biological replicates). Quantification of known SAOB, however, revealed quite similar trends in the duplicate chemostats. The explanation for this and possible impact by microbial variations on community functions and performances are unclear at this point. Similar results have been shown in previous microbial studies of parallel digesters under high‐stress, showing diverse structures at genus and species level OTUs (Goux *et al*., 2015; De Vrieze *et al*., 2016). In line with present result, these studies report on similar and stable digester performances despite microbial divergences. This highlights the challenges in interpreting links between microbial dynamics, operating conditions and process performance and emphasizes the need for increased understanding within this area to search for potential sources of variability and significances for process performances.

### Differences in richness and dynamics between mesophilic and thermophilic enrichment communities mimic trends seen in complex anaerobic communities

The initial strain richness and evenness were significantly lower in the thermophilic than in the mesophilic microbial communities, which are in line with other studies (Levén *et al*., 2007; Guo *et al*., 2014; Jang *et al*., 2016). We observed declining trends for these indices in all mesophilic and thermophilic high‐acetate chemostats throughout operation, which was expected as feeding a restricted number of substrates limits the potential metabolic pathways used for microbial growth and thus likely affects the phylogenetic distribution. Notably, the M_H_ communities were still significantly higher in richness at the end of the operating trial, indicating that mesophilic temperatures support a more diverse community than thermophilic conditions during a restricted feeding strategy.

Alpha diversity of microbial communities displayed significantly decreased richness and evenness in the M_L_ chemostats over time but, contradicting expectations, these indices remained similar at all T_L_ sampling points. However, due to the higher initial level at mesophilic temperature, similar ranges were recorded at both temperatures by the end of the trial, indicating that the low content of yeast extract and/or cysteine included in the medium was enough to support growth of a relatively diverse microbiota at both temperatures.

### Highly abundant OTUs suggest that mesophilic *Clostridiaceae* sp. and thermophilic *Syntrophaceticus* sp. are drivers of syntrophic acetate degradation

Despite the SAO‐selective conditions applied in the chemostats, previously known SAOB did not represent dominant populations. Thus, even though their abundances were higher in high‐acetate than in low‐acetate conditions (which suggests SAO activity), these SAOB were probably not the major acetate degraders in the systems investigated. However, the T‐RFLP profiling and partial *fhs* gene sequencing strongly indicated presence of bacteria previously found in high‐ammonia SAO‐dominated digesters (Westerholm *et al*., 2015; Moestedt *et al*., 2016; Müller *et al*., 2016). Unfortunately, it is currently not possible to phylogenetically position these bacteria based on the *fhs* gene. However, the Illumina results highlighted ubiquity and high relative abundance of a Clostridiaceae sp. (OTU_M_H_C) and a *Syntrophaceticus* sp. (OTU_T_H_S), which strongly suggests them as novel SAOB candidates.

The closest relatives of Clostridiaceae sp. OTU_M_H_C (*A. halophilus* and *A. oremlandii*) are moderately halophilic (optimum growth at pH 8) and fermentative bacteria (Fisher *et al*., 2008; Wu *et al*., 2010). Some characteristics of these *Alkaliphilus* species resemble the features of SAOB when grown in pure culture, such as similar substrate patterns, formation of formate and acetate as main fermentation products (*A. halophilus*) and respiratory capabilities (Schnürer *et al*., 1996; Hattori *et al*., 2000; Fisher *et al*., 2008; Westerholm *et al*., 2010, 2011a,b; Wu *et al*., 2010). *A. oremlandii* also oxidizes acetate, with reduction of thiosulfate as the electron acceptor (Fisher *et al*., 2008), which is a capability it shares with the SAOB *T. phaeum* (Hattori *et al*., 2000). *Alkaliphilus* species have also been detected in high‐ammonia biogas digesters (> 0.3 g NH_3_‐N l^−1^/8–10 g NH^+^_4_‐N l^−1^, Kovács *et al*., 2013; Müller *et al*., 2016; Tsapekos *et al*., 2017; Ziganshina *et al*., 2017), suggesting that they play a critical role in biogas digesters operating under such conditions. Their presence has been attributed to the ability of certain members to encode crucial peptidases for proteolysis of proteins (Stolze *et al*., 2015). Moreover, the levels of these bacteria have been shown to correlate with an ammonia‐induced shift from aceticlastic methanogenesis to SAO (Müller *et al*., 2016). Phylogenetic analyses of the deduced FTHFS amino acid sequences positioned retrieved sequences (represented by the T‐RFs 58 and 108 bp) close to *fhs* genes of *Alkaliphilus* sp., which indicates Clostridiaceae sp. OTU_M_H_C. Other sequences assigned to the T‐RF 406 bp significantly increased in abundance during operation of both M_H_ chemostats, positioned close to the known SAOB *P. lettingae* in the phylogenetic analyses and are thus considered interesting SAOB candidates.

The taxonomic survey of the OTU_T_H_S, which represented a significant proportion of the bacterial community in the thermophilic T_H_ chemostats, revealed phylogenetic relatedness (97% identity) with the cultured representative of *S. schinkii*. The mesophilic *S. schinkii* oxidizes acetate in association with the hydrogenotrophic *M. bourgensis*, tolerates high ammonium levels and has a growth temperature ranging from 25 to 40°C (Westerholm *et al*., 2010). Despite the narrow temperature range for growth of this type strain, relatives to this species have been found in mesophilic biogas digesters (37–40°C; Westerholm *et al*., 2011a,b, 2012a,b, 2015; Karlsson *et al*., 2012; Moestedt *et al*., 2014; Sun *et al*., 2014; Müller *et al*., 2016) and in digesters operating at moderate (42–45°C; Moestedt *et al*., 2014; Westerholm *et al*., 2015) and thermophilic (49–60°C; Weiss *et al*., 2008; Sun *et al*., 2014; Lebuhn *et al*., 2015; Müller *et al*., 2016) temperatures. Together, these findings indicate that, apart from the wide‐ranging temperature span, relatives to *S. schinkii* are able to remain active under varying operating conditions in terms of ammonia concentration, HRT, substrate feed and digester configuration. Despite the lower phylogenetic relationship between OTU_T_H_S and *T. phaeum*, the thermophilic growth condition was a shared capability of these species. Searches in the Blast database furthermore revealed that sequences with identical identity to OTU_T_H_S have been discovered in syntrophic propionate‐degrading communities (Sugihara *et al*., 2007), indicating ability to degrade both propionate and acetate or involvement in acetate degradation in association with syntrophic propionate‐degrading bacteria. 16S rRNA gene sequences identical to OTU_T_H_S have previously also been found in thermophilic (53°C) dry anaerobic digestion of waste paper‐based medium and suggested to be involved in SAO (OTU 1‐1B‐29 in Tang *et al*., 2011) The *fhs* gene sequencing indicated that the gene from OTU_T_H_S was not targeted by the current primers. Hence, further development of the fhs‐primers targeting thermophilic species is needed for a more complete coverage of potential SAOB. However, the significantly higher relative abundance of OTU_T_H_S in T_H_ than in T_L_, the relatively close identity to the SAOB *S. schinkii* and *T. phaeum* and the detection of genes with high identity in systems with a high possibility of being SAO‐dominated indicate that OTU_T_H_S may be a hitherto undescribed species in the genus *Syntrophaceticus* that is capable of SAO.

### The strict hydrogenotrophs *M. bourgensis* and Methanothermobacter are likely SAO partners, but may compete with formate‐utilizing bacteria

The dominance of the strictly hydrogenotrophic *M. bourgensis* and *Methanothermobacter* in the high‐acetate chemostats strongly suggests SAOB partnerships. The *mcrA* gene analysis for M_H_ also suggested dominance of a *M. bourgensis* strain previously found in a biogas digester supplied with trace elements (OTU15 in Westerholm *et al*., 2015) that was the inoculum source for our mesophilic enrichment chemostats. This parental biogas digester had relatively high richness of *mcrA* genes (~14 dominant OTUs with relatively even distribution, Westerholm *et al*., 2015), and the dominance of one particular strain in the M_H_ chemostats was thus somewhat surprising. A relevant finding in the previous study is that OTU15 was not detected in a digester operating under corresponding conditions, which did not receive trace elements (Westerholm *et al*., 2015). This indicates that continuous feeding of the trace element‐rich medium in the M_H_ chemostats may have been particularly advantageous for this *M. bourgensis* strain. Relatively low partial hydrogen partial pressure was another differing operating parameter between the parental digester and other digesters in the previous study (Westerholm *et al*., 2015). While the influence of this particular strain on hydrogen removal remains to be investigated, the ability for hydrogen removal to low levels (due to its high affinity for hydrogen) and the tolerance to high ammonia levels have been suggested as key drivers for *M. bourgensis* suitability as a SAO methanogenic partner (Westerholm *et al*., 2015; Neubeck *et al*., 2016). This is also stressed by the frequent detection of *M. bourgensis* in SAO‐dominated biogas digesters (reviewed in Westerholm *et al*., 2016).

In thermophilic SAO digesters, Methanomicrobiales and Methanobacteriales (*Methanothermobacter*) are frequently reported as the dominant hydrogenotrophic methanogens (reviewed in Westerholm *et al*., 2016). Similarly, the Illumina results in the present study demonstrated high relative abundance of *Methanothermobacter*. Methanobacteriales was also detected by qPCR analyses of the T_H_ chemostats, but corresponding analyses for Methanomicrobiales indicated that it was present in considerably higher abundance in these chemostats. These conflicting findings obtained in Illumina sequencing and specific qPCR analyses of ratios between Methanomicrobiales and Methanobacteriales can be related to differences in specificity in the primers used for these analyses. Due to the need for high sensitivity, encompassing as many 16S rRNA variants as possible, the Illumina primers will likely not have the same specificity for archaeal methanogens as the qPCR primers.

The relatively high abundances of *Sulfurospirillum* sp. in M_H_ and *Bacillus* sp. in the T_H_ chemostats were puzzling, as SAOB candidates have not previously been suggested to belong to these genera. However, negligible detection of these OTUs in M_L_ and T_L_ indicates involvement in acetate degradation. The strict anaerobic requirements and temperature ranges for growth reported for the isolated type strains of the closest relatives, that is *S. alkalitolerans* and *B. infernus*, are in agreement with the conditions in M_H_ and T_H2_. Furthermore, the substrate patterns of these bacteria resemble what has been observed in pure cultures of known SAOB (e.g. lactate, pyruvate, fumarate or glucose, Boone *et al*., 1995; Schnürer *et al*., 1996; Hattori *et al*., 2000; Balk *et al*., 2002; Westerholm *et al*., 2010, 2011a,b; Sorokin *et al*., 2013). However, *S. alkalitolerans* is also capable of utilizing formate and H_2_ (with acetate as carbon source, Sorokin *et al*., 2013), and all components for growth of this bacterium would thus be provided in the present acetate‐enriched chemostats, with formate or H_2_ produced by SAOB (Müller *et al*., 2015) and acetate present in the medium. *B. infernus* can also use formate with Fe(III), MnO_2_, trimethylamine oxide or nitrate as an electron acceptor, but cannot grow on acetate (Boone *et al*., 1995). Hence, these data indicate that growth of *B. infernus* and *Sulfurospirillum* sp. may rely on supply of formate and/or H_2_ from acetate oxidation by SAOB. This would thus lead to competition between *B. infernus* and *Sulfurospirillum* sp. and the hydrogenotrophic methanogens for substrate, although further investigation is required to establish such a scenario. However, despite the uncertain metabolic roles of *Sulfurospirillum* OTU_M_H_Ep and *B. infernus* in our enrichment chemostats, their detection confirms them to be ammonia‐tolerant.

To conclude, the continuous enrichment approach applied in the present study permitted analysis of a community specialized in acetate conversion, with a limited confounding effect of other metabolic groups. As anticipated, the populations identified as dominant SAOB were present at very low abundances or below detection in the initial operating period. The continuous acetate feeding then strongly restricted the microbial community structure and decreased phylotype richness, which enabled detection of potential SAOB after a few HRT. Based on our results, we posit SAO capability of the highly abundant OTU_M_H_C (GenBank accession number MG356789) and OTU_T_H_S (GenBank accession number MG356790) in mesophilic and thermophilic conditions respectively. However, this requires further investigation. Considering the importance of acetate removal for the overall biogas production system, identifying acetate‐degrading strains capable of remaining active in the presence of high ammonia concentrations is of the utmost importance. Whether bioaugmentation of key SAO populations or/and altered operating conditions to support their activity is a suitable approach to improve biogas yield remains to be determined. Nonetheless, this is an area in which increased insights into how to predict behaviours of key microbial populations and support their activity shows great promise for maintaining robust performance under stressed conditions.

## Experimental procedures

### Anaerobic chemostat set‐up and operation

Four identical laboratory‐scale continuously stirred (80 rpm) tank chemostats (Belach Bioteknik, Stockholm, Sweden) with working volume 1.1 l were operated in parallel in continuous mode under two temperature regimes; mesophilic (M, 37°C) and thermophilic (T, 52°C). Anoxic and sterile bicarbonate‐buffered basal medium (BM, Westerholm *et al*., 2010) supplemented with 16.1 g NH_4_Cl l^−1^ and 0.4 g sodium acetate l^−1^ (mesophilic: M_L1_, M_L2_ thermophilic: T_L1_, T_L2_) or 7.5 g sodium acetate l^−1^ (mesophilic: M_H1_, M_H2_; thermophilic: T_H1_, T_H2_) was fed to the chemostats using four separate peristaltic pumps (Belach Bioteknik) at a speed of 26 μl/min. The chemostat compartments were placed on balance, which activated the outflow pumping when required, to maintain a hydraulic retention time (HRT) of 28 days. Each operating set was maintained for about 160 days (> 6 retention times).

The chemostats were inoculated with 1 l sterile BM and 0.1 l sludge during flushing with N_2_. The M_H_ and M_L_ chemostats were inoculated with sludge from a mesophilic high‐ammonia (5.4 g NH_4_^+^‐N l^−1^, 0.6–0.9 g NH_3_ l^−1^, Table S6) digester degrading food waste and albumin, and T_H_ and T_L_ were started with sludge from a thermophilic (52°C) commercial‐scale biogas plant degrading industrial food waste and manure (Kungsängens gård, Uppsala, Sweden, 3.2 g NH_4_^+^‐N l^−1^, 0.6 g NH_3_ l^−1^, Table S6). Both these digesters have been shown in previous studies to have SAO as the dominant acetate‐degrading pathway and to include high abundance of known SAOB (digesters D^TE^37 and M; (Sun *et al*., 2014; Westerholm *et al*., 2015; Müller *et al*., 2016).

### Analytical investigations

Volatile fatty acid (VFA) concentrations (acetate, propionate, butyrate, isobutyrate, valerate, isovalerate, capronate and isocapronate) were analysed continuously using high‐performance liquid chromatography (HPLC), and methane content in the gas was determined by gas chromatography (GC) as described previously (Westerholm *et al*., 2012a,b).

### DNA extraction, quantitative PCR, sequencing of the mcrA gene, terminal restriction fragment length polymorphism (T‐RFLP) analyses, cloning and phylogenetic analysis of partial fhs gene

Samples for molecular analyses were collected from each chemostat on a weekly basis and stored at ‐20°C until further use. DNA was extracted using the FastDNA soil kit (MP Biomedicals, France). Three DNA extracts from each time point and chemostat were included in the molecular analyses, to obtain three technical replicates per biological replicate (the two parallel chemostats).

To obtain information on absolute abundances of known SAOB (*C. ultunense*,* T. phaeum*,* S. schinkii* and *T. acetatoxydans*), 16S rRNA gene‐targeting qPCR assays were conducted as described previously (Westerholm *et al*., 2011a,b; Sun *et al*., 2014). The primer sets MBT and MMB (Yu *et al*., 2005) were used for targeting species of Methanobacteriales and Methanomicrobiales respectively. Descriptions of primer pair *fhs*OTU3‐*fhs*OTU10, targeting the *fhs* gene (encoding a key enzyme in acetogenic and SAO metabolism) of potential SAOB, and the qPCR protocol can be found in Müller *et al*. (2016).

To further investigate the methanogenic communities, PCR amplification with primers mlas and mcrA‐rev was used in recommended reaction conditions (Steinberg and Regan, 2008). The amplicons were then sequenced with mcrA‐rev primer (Macrogen, Seoul, Korea). Quality check, editing and sequence assembly were performed with Geneious v10.1.3 (Kearse *et al*., 2012).

Terminal restriction fragment length polymorphism (T‐RFLP) was used to characterize acetogenic communities in the enrichment chemostats using the degenerated primer pair designed by Müller *et al*. (2012), which was slightly modified: 3‐SAO*fhs*‐fw (CCNACNCCNNNNGGNGANGGNAA) and 3‐SAO‐rev (ATITTIGCIAAIGGNCCNSCNTG). The *fhs* gene T‐RFLP data sets were collected as triplicates from one sampling point in each HRT in the high‐acetate chemostats and on two occasions in the low‐acetate chemostats. The protocol described in Müller *et al*.(2012) was used for the analyses, with the exception that AluI (New England BioLabs, Ipswich, MA, USA) was used for digestion of PCR products. The sample from chemostat T_L1_ HRT 6 did not fulfil the filtering criteria and was thus excluded from the analysis. The procedures in Müller *et al*.(2016) were followed to construct *fhs* clone libraries (from M_H_ and T_H_ chemostats at HRT 6) and to analyse and edit retrieved sequences. Multiple sequences alignment of the deduced amino acid *fhs* sequences and references sequences from NCBI (indicated accession numbers in Fig. 2) was executed with MAFFT v7.245 (Katoh and Standley, 2013) command line environment in 1000 iteration. The Maximum likelihood phylogenetic tree with 1000 bootstrap was constructed in the FastTree Version 2.1.8 SSE3 (Price *et al*., 2010) command line using the Whelan‐And‐Goldman 2001 model on the resulted MAFFT protein alignment. MAFFT and FastTree modules are available on the SLU Global Bioinformatics Servers described elsewhere in this article. All *fhs* sequence data reported in this study were deposited in the NCBI GenBank database (http://www.ncbi.nlm.nih.gov/genbank/) under the accession numbers MG012806–MG012840 and MG385064–MG385074 for M_H_ and T_H_ chemostats respectively.

### 16S rRNA gene Illumina sequencing

Construction of 16S amplicon libraries using primers 515′F (GTGBCAGCMGCCGCGGTAA, Hugerth *et al*., 2014) and 805R (GACTACHVGGGTATCTAATCC, Herlemann *et al*., 2011) and Illumina sequencing of 16S rRNA genes was carried out in triplicate on DNA extracts from each time point and chemostat as described by Müller *et al*. (2016). Paired‐end sequencing was performed on an Illumina MiSeq instrument at SciLifeLab Stockholm, Sweden.

The resulting sequence analysis was performed on the Global Bioinformatics Servers at SLU, Uppsala, Sweden, running CentOS Linux release 7.1.1503, with module handling by Modules based on Lua: Version 6.0.1. The modules loaded were Cutadapt 1.13, Sickle 1.0 and Mothur 1.39.5. Raw data were trimmed using Cutadapt followed by removal of singletons and low quality reads by Sickle (Joshi and Fass, 2011; Martin, 2011; Schloss *et al*., 2012). Data were then processed using Mothur, following the guidelines of the Mothur MiSeq SOP with slight modifications (Kozich *et al*., 2013); pcr.seqs was performed using the custom primers utilized in amplification of the 16S rRNA gene, while the database used was the Mothur‐formatted SILVA. Full‐length sequences and taxonomy references were taken from the database release 128. Classification was performed using classify.seqs and the 16S rRNA reference (RDP) database, version 16. As no mock samples were processed in parallel with the other samples, no assessment of error rates was performed. The full history from the mother run and scripts can be found in Appendix S1. For sequence data and statistics of analysis, see Table S7.

Changes in phylogenetic diversity over time were tested using regression analyses in R. The MiSeq data are available at the National Center for Biotechnology Information Sequence Read Archive (accession PRJEB21746). Representative sequences of potential SAOB candidates were compared with publicly available sequences using the Basic Local Alignment Search Tool (BLAST) algorithm (Altschul *et al*., 1990) provided by the National Center for Biotechnology Information (NCBI, http://www.ncbi.nlm.nih.gov). The representative partial 16S rRNA gene sequences for OTU_M_H_C and OTU_T_H_S were deposited in the GenBank database (http://www.ncbi.nlm.nih.gov/genbank/) under the accession numbers MG356789 and MG356790 respectively.

### Statistical analysis

The statistical significance of differences between low‐ and high‐acetate chemostats, and over time in the chemostats, was determined using analysis of variance (ANOVA) in R Studio version software (http://www.r-project.org, Team R, 2016). NMDS analysis was carried out on the sample‐OTU matrix using the Bray–Curtis distances (R package Vegan; Oksanen *et al*., 2015).

## Conflict of interest

None declared.

## Supporting information


**Fig. S1.** Acetate concentration in the high‐acetate chemostats (fed medium containing 7.5 g acetate l^−1^) operating at 37°C (A) and 52°C (B).
**Fig. S2.** Rarefaction curves generated from OTUs at 3% sequence dissimilarity occuring in the four mesophilic and the four thermophilic chemostats operated in the present study.
**Fig. S3.** Average richness (number of OTUs, Chao) and Shannon Diversity (H') of microbial communities in (A) mesophilic and (B) thermophilic temperature conditions.
**Fig. S4.** Non‐metric multidimensional scaling (NMDS) analysis of the Bray‐Curtis dissimilarity index of the microbial community OTUs (≥ 97% identity) based on Illumina sequencing of 16S rRNA genes in mesophilic (A) and thermophilic (B) chemostats fed 7.5 (M_H_/T_H_) or 0.4 g acetate l^−1^ (M_L_/T_L_).
**Fig. S5.** *fhs* (formyltetrahydrofolate synthetase) gene profiling by means of T‐RFLP in mesophilic (A) and thermophilic (B) chemostats fed 7.5 (M_H_/T_H_) and 0.4 (M_L_/T_L_) g acetate l^−1^.
**Fig. S6.** Average *fhs* gene copies obtained in quantitative PCR (qPCR) analyses targeting the *fhs* gene of potential SAOB in mesophilic chemostats fed high (M_H_, 7.5 g l^−1^) and low (M_L_, 0.4 g l^−1^) acetate.
**Fig. S7.** Total bacteria abundance per mL sludge in (A) mesophilic and (B) thermophilic chemostats.
**Table S1.** Summary of operating conditions of duplicate mesophilic (M) and thermophilic (T) chemostats receiving high‐acetate (_H_) or low‐acetate feed (_L_). The values are mean of > 3 analyses and standard error of the mean (SEM).
**Table S2.** Significant differences over time and between mesophilic high‐acetate (M_H_, 7.5 g l^−1^) and low‐acetate (M_L_, 0.4 g l^‐1^) chemostats in quantitative PCR (qPCR) data and diversity indices.
**Table S3.** Significant differences in quantitative PCR (qPCR) data and diversity indices between thermophilic (52°C) chemostats fed high (T_H_, 7.5 g l^−1^) and low (T_L_, 0.4 g l^−1^) acetate and over time.
**Table S4.** Significant differences in acetate levels, richness and evenness indices between mesophilic (M) and thermophilic (T) high‐acetate (_H_, 7.5 g l^−1^) chemostats.
**Table S5.** Accession numbers of *fhs* sequences retrived in clone libraries from the chemostats M_H1_ M_H2_, T_H1_ and T_H2_ after 6 hydraulic retention times of operation (HRT 28 days).
**Table S6.** Operating conditions for the two biogas digesters that were the inoculum source for the mesophilic (37°C) and thermophilic (52°C) chemostats in the present study.
**Table S7.** Sample name in Illumina sequencing data, sequencing results, source chemostat and the hydraulic retention time (HRT) for sampling.
**Appendix S1.** Scripts used for processing sequence reads in 16S rRNA analysis.
