# Supplementary material for: Detection of novel syntrophic acetate‐oxidizing bacteria from biogas processes by continuous acetate enrichment approaches
Source: Microb Biotechnol. 2017 Dec 4;11(4):680–93. doi: 10.1111/1751-7915.13035 (PMC6011928; doi:10.1111/1751-7915.13035)
Supplement: Supplementary file 1 — Fig. S1. Acetate concentration in the high‐acetate chemostats (fed medium containing 7.5 g acetate l−1) operating at 37°C (A) and 52°C (B). Fig. S2. Rarefaction curves generated from OTUs at 3% sequence dissimilarity occuring in the four mesophilic and the four thermophilic chemostats operated in the present study. Fig. S3. Average richness (number of OTUs, Chao) and Shannon Diversity (H') of microbial communities in (A) mesophilic and (B) thermophilic temperature conditions. Fig. S4. Non‐metric multidimensional scaling (NMDS) analysis of the Bray‐Curtis dissimilarity index of the microbial community OTUs (≥ 97% identity) based on Illumina sequencing of 16S rRNA genes in mesophilic (A) and thermophilic (B) chemostats fed 7.5 (MH/TH) or 0.4 g acetate l−1 (ML/TL). Fig. S5. fhs (formyltetrahydrofolate synthetase) gene profiling by means of T‐RFLP in mesophilic (A) and thermophilic (B) chemostats fed 7.5 (MH/TH) and 0.4 (ML/TL) g acetate l−1. Fig. S6. Average fhs gene copies obtained in quantitative PCR (qPCR) analyses targeting the fhs gene of potential SAOB in mesophilic chemostats fed high (MH, 7.5 g l−1) and low (ML, 0.4 g l−1) acetate. Fig. S7. Total bacteria abundance per mL sludge in (A) mesophilic and (B) thermophilic chemostats. Table S1. Summary of operating conditions of duplicate mesophilic (M) and thermophilic (T) chemostats receiving high‐acetate (H) or low‐acetate feed (L). The values are mean of > 3 analyses and standard error of the mean (SEM). Table S2. Significant differences over time and between mesophilic high‐acetate (MH, 7.5 g l−1) and low‐acetate (ML, 0.4 g l‐1) chemostats in quantitative PCR (qPCR) data and diversity indices. Table S3. Significant differences in quantitative PCR (qPCR) data and diversity indices between thermophilic (52°C) chemostats fed high (TH, 7.5 g l−1) and low (TL, 0.4 g l−1) acetate and over time. Table S4. Significant differences in acetate levels, richness and evenness indices between mesophilic (M) and thermophilic (T [file MBT2-11-680-s001.docx]

**Supporting Information**

**Fig. S1.**

**A B**

**
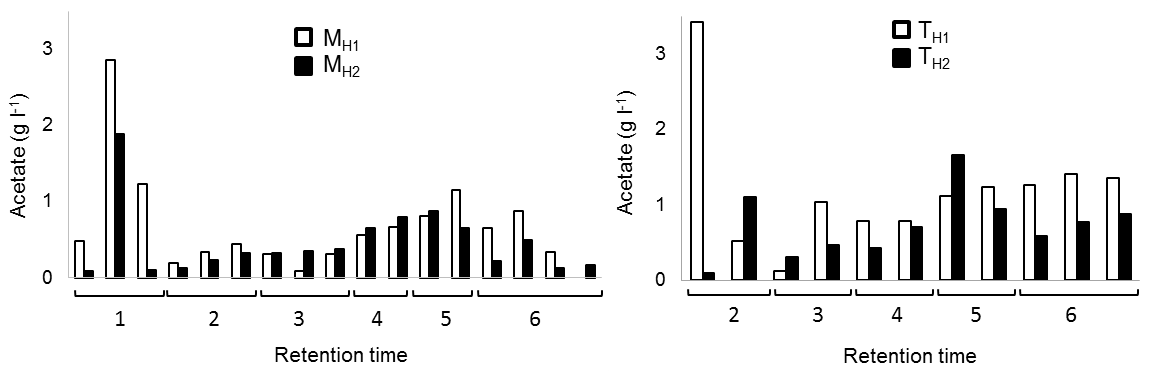
**

**Fig. S1.** Acetate concentration in the high-acetate chemostats (fed medium containing 7.5 g acetate L^-1^) operating at 37°C (A) and 52°C (B). The chemostats were operated in parallel to obtain biological duplicates. Each retention time represent 28 days of operation.

**Fig. S2**

**
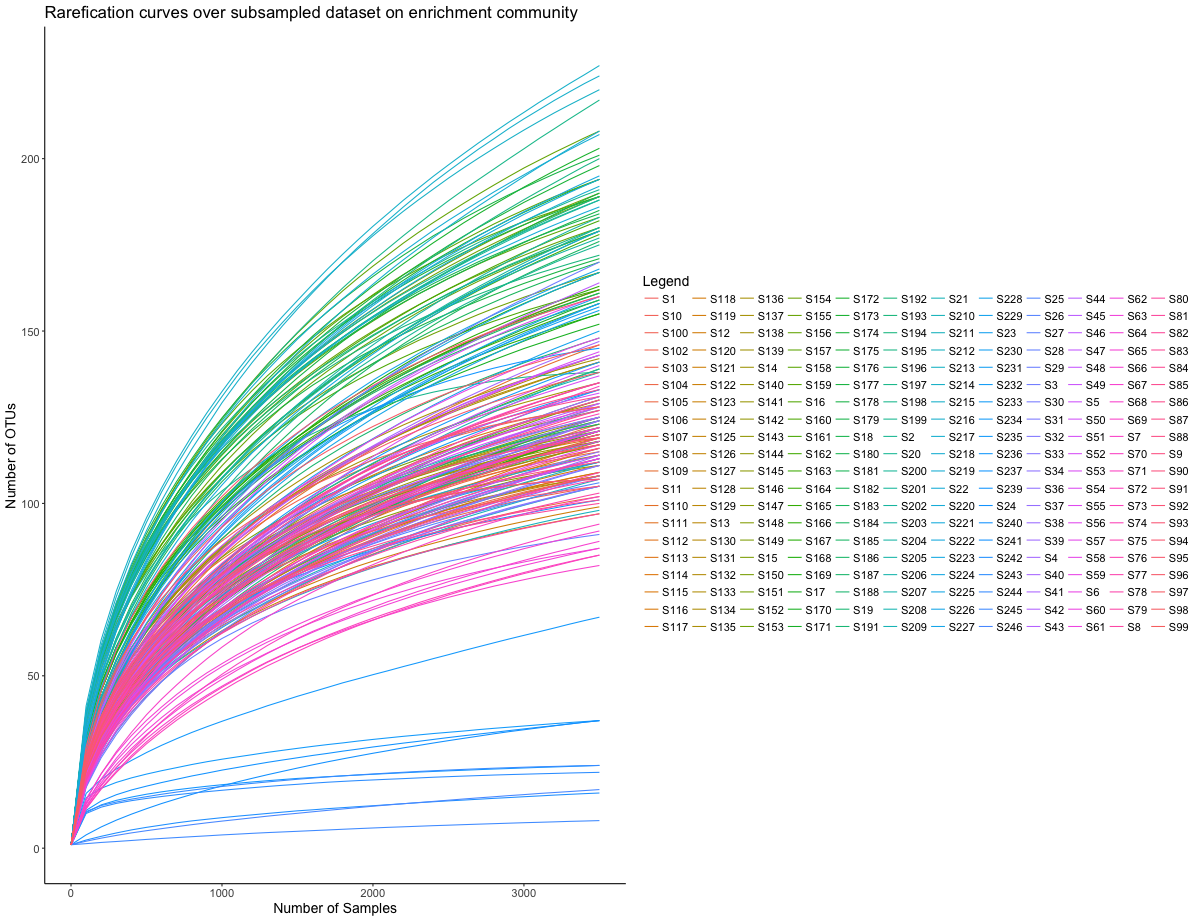
**

**Fig. S2.** Rarefaction curves generated from OTUs at 3% sequence dissimilarity occuring in the four mesophilic and the four thermophilic chemostats operated in the present study. The two chemostats were run in parallel and continuously fed medium containing 0.4 g L^-1^ (_L_) or 7.5 g L^-1^ (_H_) acetate. Data were obtained from triplicate Illumina MiSeq sequencing runs from 28 sampling points in the mesophilic and 48 sampling points in the thermophilic chemostats. Chemostat and sampling point for the samples are found in Table S7.

**Fig. S3.**

**
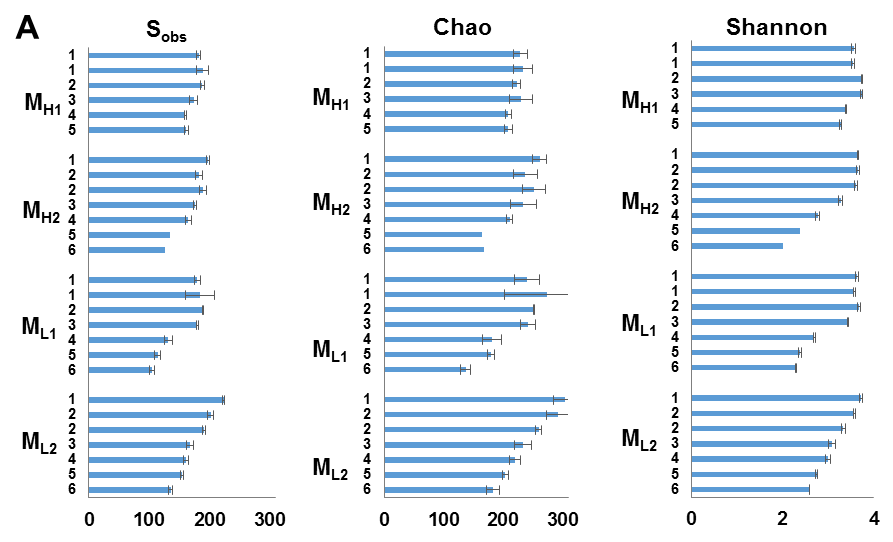

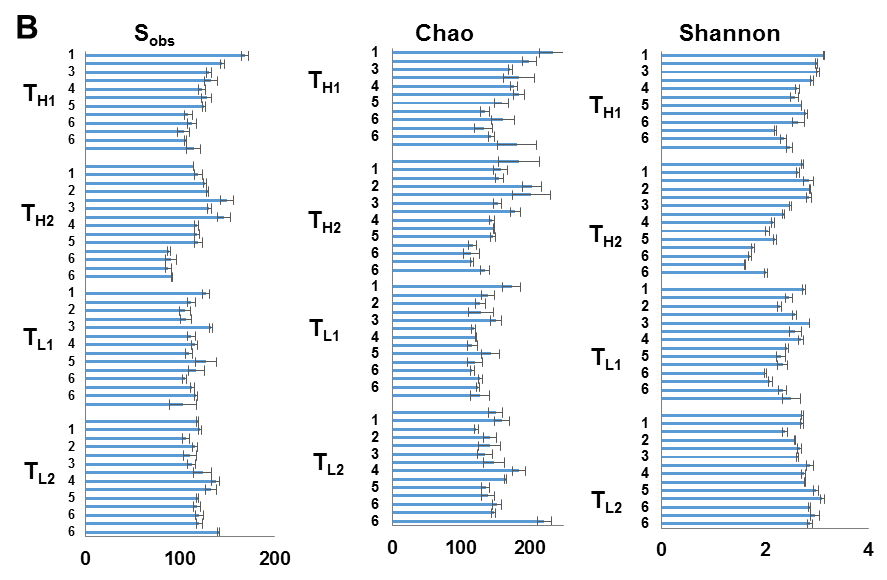
**

**Fig. S3.** Average richness (number of OTUs, Chao) and Shannon Diversity (H’) of microbial communities in (A) mesophilic and (B) thermophilic temperature conditions. Chemostat affiliation (M mesophilic, T thermophilic, _H_ high-acetate, _L_ low-acetate) and the hydraulic retention time at the time of sampling are shown along the left side of the diagram. Error bars represent standard error of the mean (SEM) of triplicate Illumina sequencing and calculations were performed at 97% sequence identity.

**Fig. S4**

**A B**

**
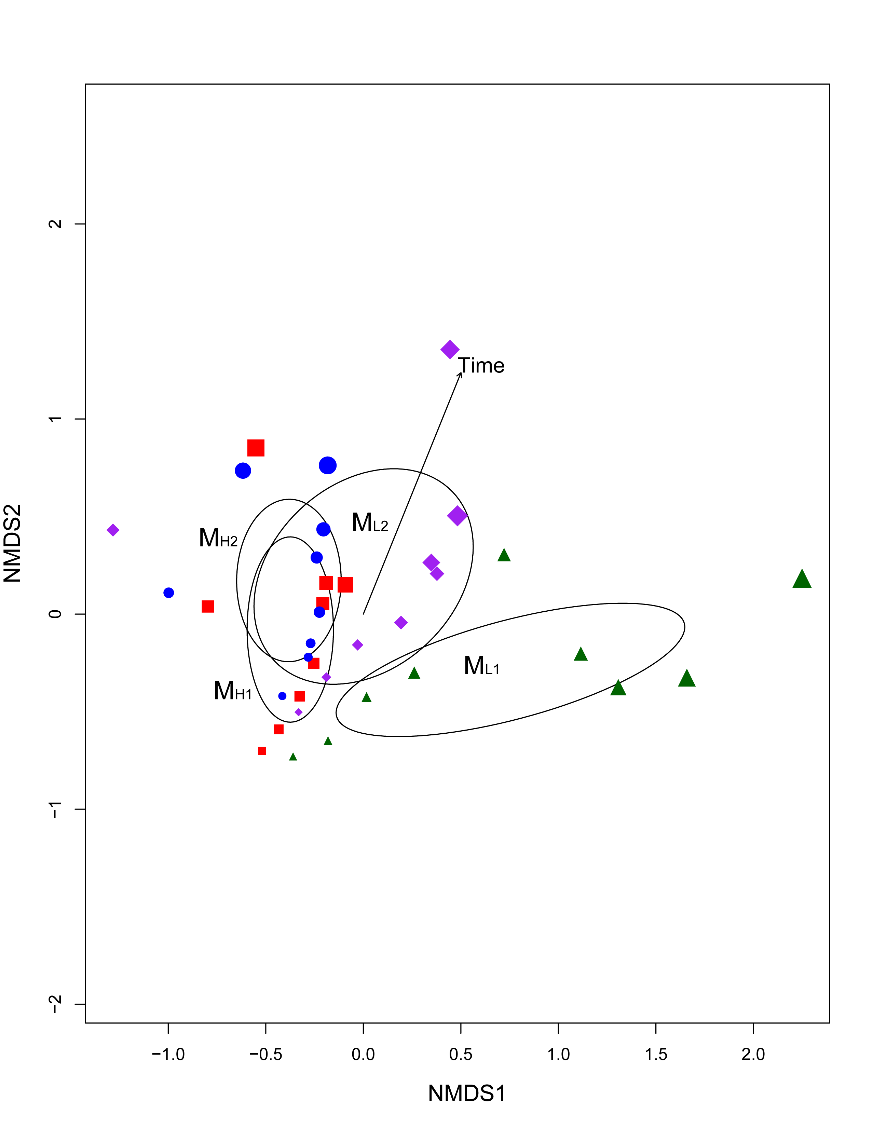

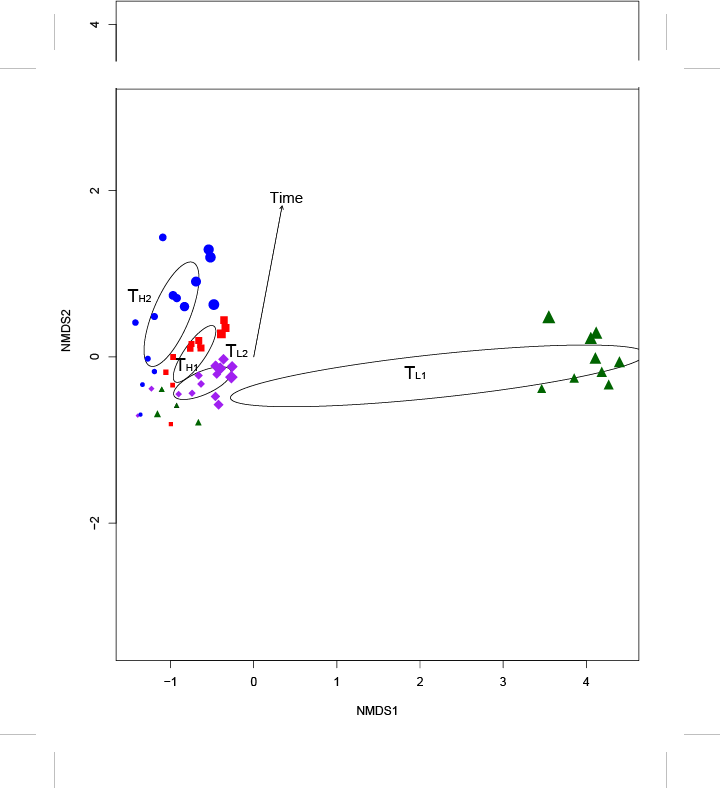
**

**Fig. S4.** Non-metric multidimensional scaling (NMDS) analysis of the Bray-Curtis dissimilarity index of the microbial community OTUs (≥ 97% identity) based on Illumina sequencing of 16S rRNA genes in mesophilic (A) and thermophilic (B) chemostats fed 7.5 (M_H_/T_H_) or 0.4 g acetate L^-1^ (M_L_/T_L_). The samples were taken at 9-14 time points from each chemostat and samples are symbolised by a single point coloured and shaped by chemostat. The size of the point increases with time. Environmental fitting was performed to correlate chemostat and time to the community structure. The arrow represents the effect of time showing the direction of the (increasing) gradient, while the length is proportional to the correlation between variable and the ordination. Using two dimensions (k=2), stress values of 0.133 and 0.0759 were obtained from mesophilic and thermophilic chemostats, respectively

**Fig. S5**

**A**

**
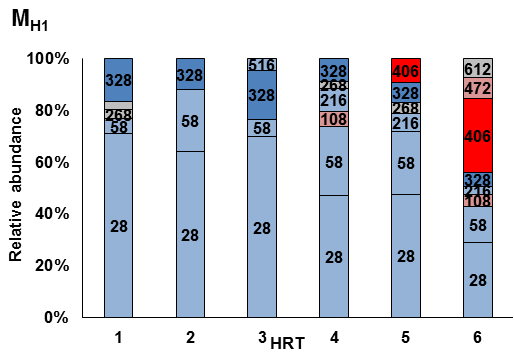

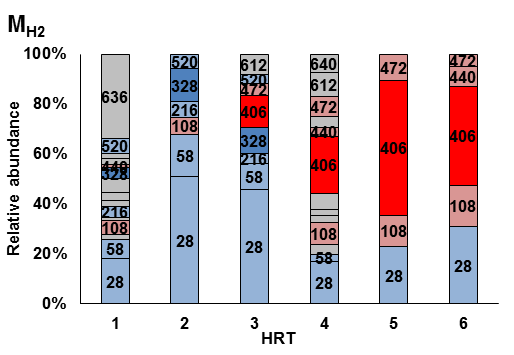

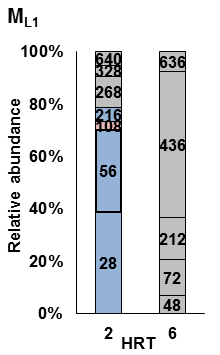

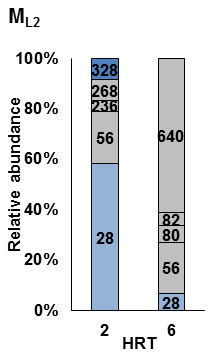
**

**B**


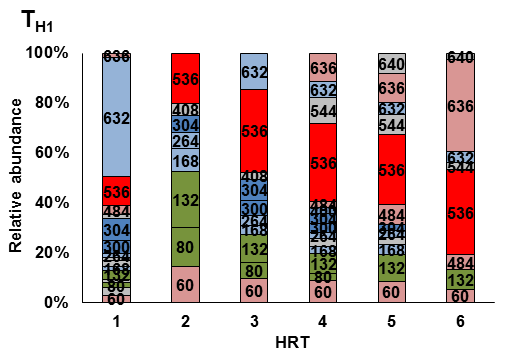

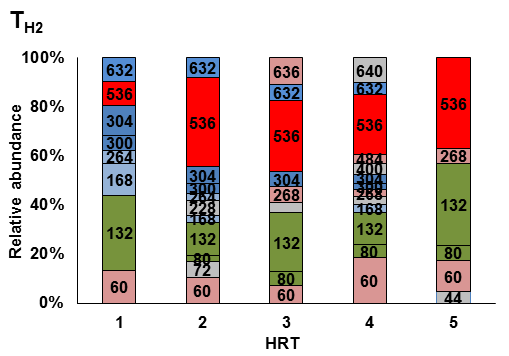

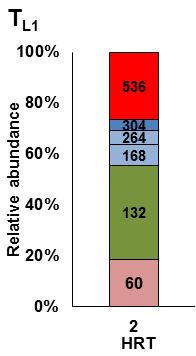

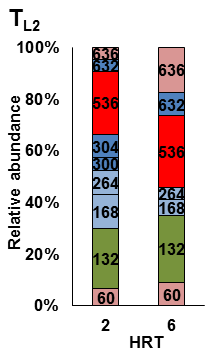


**Fig. S5.** *fhs* (formyltetrahydrofolate synthetase) gene profiling by means of T-RFLP in mesophilic (A) and thermophilic (B) chemostats fed 7.5 (M_H_/T_H_) and 0.4 (M_L_/T_L_) g acetate L^-1^. The hydraulic retention time (HRT 28 days) at the time of sampling is displayed on the x-axis. Dark blue and dark red bars represent fragments that significantly (P<0.05) decreased and increased in relative abundance over time in both high-acetate chemostats, respectively, while lighter tones indicate significance in one of the duplicate chemostats. Grey bars indicate fragments appearing occasionally and green bars are fragments that showed stable (132 bp) or contradictory trends (80 bp) in the replicate thermophilic chemostats. Bars show average values of triplicate analyses (except for duplicate samples in M_H1_ HRT 5 and 6 and single sample in M_L1_ HRT 5 and T_L2_ HRT 2). Due to experimental failure, the samples from T_H2_ and T_L1_ at HRT 6 were excluded from the analysis. If assigned as 635-640 bp in the T-RFLP, the corresponding sequence indicated no restriction site for *Alu*1.

**Fig. S6**


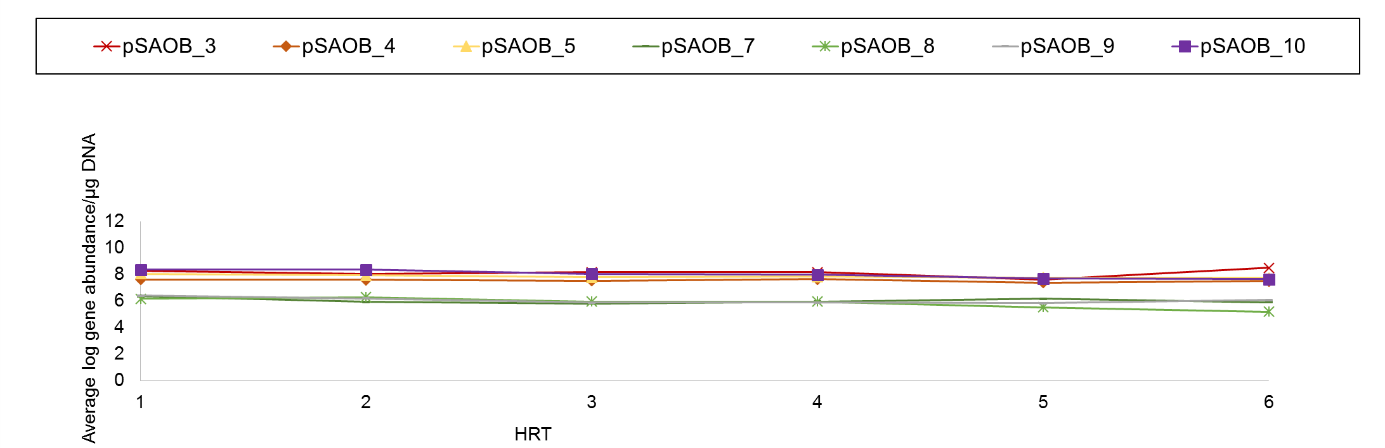


**
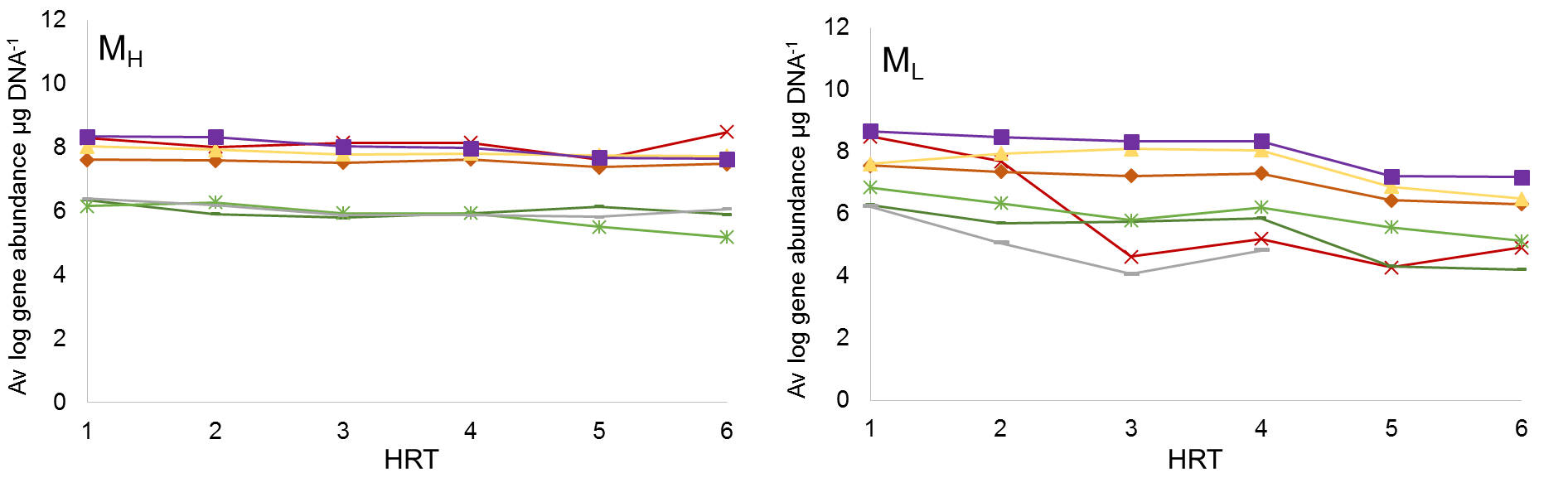
**

**Fig. S6.** Average *fhs* gene copies obtained in quantitative PCR (qPCR) analyses targeting the *fhs* gene of potential SAOB in mesophilic chemostats fed high (M_H_, 7.5 g L^-1^) and low (M_L_, 0.4 g L^-1^) acetate. Average values from the duplicate chemostats are given for samples taken after operation for 1-6 hydraulic retention times (HRT 28 days, y-axis).

**Fig. S7.**

**
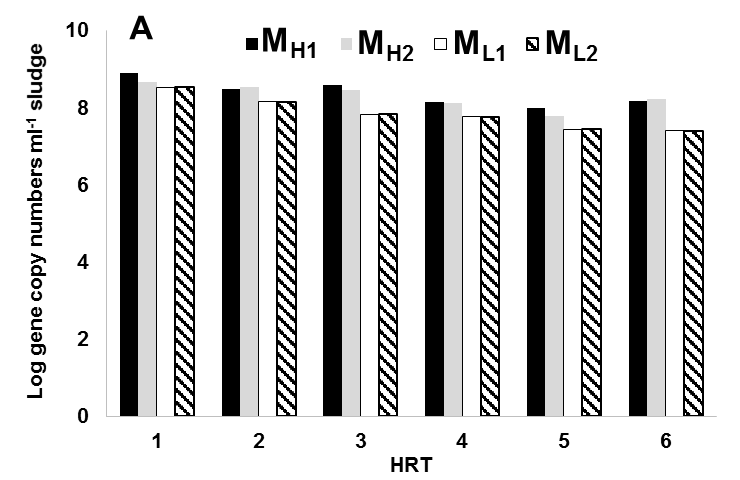

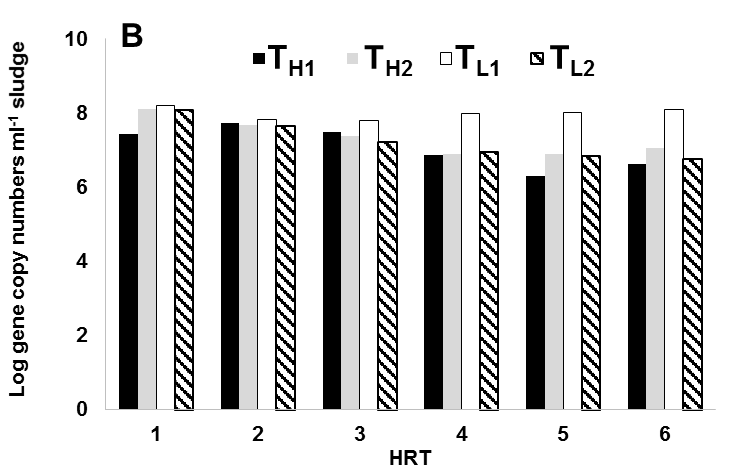
**

**Fig. S7.** Total bacteria abundance per mL sludge in (A) mesophilic and (B) thermophilic chemostats. The hydraulic retention time (HRT 28 days) at the point of sampling is given on the x-axis.

**Table S1.** Summary of operating conditions of duplicate mesophilic (M) and thermophilic (T) chemostats receiving high-acetate (_H_) or low-acetate feed (_L_). The values are mean of >3 analyses and standard error of the mean (SEM)

| Temp. | Chemostat | Acetate influent (g L^-1^) | Average acetate effluent (range, g L^-1^) | pH | Ammonia conc. (g NH_3_  -N L^-1^) | CH_4_ content (%) | |  |
| --- | --- | --- | --- | --- | --- | --- | --- | --- |
| 37°C | M_L1_ | 0.4 | >0.1 (>0.1-0.2) | 7.7 ± 0.1 | 0.27 ± 0.01 | 2 ± 2 |  | |
|  | M_L2_ | 0.4 | >0.1 (>0.1-0.1) | 7.8 ± 0.1 | 0.35 ± 0.05 | 9 ± 6 |  | |
|  | M_H1_ | 7.5 | 0.6 ± 0.3 (0.1-1.2) | 8.2 ± 0.1 | 0.77 ± 0.04 | 69 ± 9 |  | |
|  | M_H2_ | 7.5 | 0.4 ± 0.3 (0.1-0.9) | 8.1 ± 0.2 | 0.67 ± 0.06 | 64 ± 6 |  | |
| 52°C | T_L1_ | 0.4 | >0.1 (>0.1) | 7.7 ± 0.1 | 0.77 ± 0.09 | 0.3 ± 0.5 |  | |
|  | T_L2_ | 0.4 | >0.1 (>0.1) | 7.8 ± 0.1 | 0.95 ± 0.12 | 4 ± 2 |  | |
|  | T_H1_ | 7.5 | 1.0 ± 0.4 (0.1-1.4) | 8.1 ± 0.1 | 1.51 ± 0.08 | 69 ± 7 |  | |
|  | T_H2_ | 7.5 | 0.8 ± 0.4 (0.3-1.6) | 8.2 ± 0.1 | 1.81 ± 0.15 | 66 ± 2 |  | |

**Table S2.** Significant differences over time and between mesophilic high-acetate (M_H_, 7.5 g L^-1^) and low-acetate (M_L_, 0.4 g L^-1^) chemostats in quantitative PCR (qPCR) data and diversity indices. In chemostat comparisons, data from HRT 2-6 were included and in analysis of T-RFLP data fragments <2% at least two sample points were considered. Statistical analyses were conducted using the General Linear Model and significance was set to *P*<0.05

|  | **Difference** | **F-value** | **P** |
| --- | --- | --- | --- |
| Methanomicrobiales | M_L_ < M_H_ | 52.98 | <0.001 |
|  | Increase over time in M_H_ | 5.06 | 0.044 |
| Total bacteria (mL^-1^ sludge) | M_L_ < M_H_ | 4.5 | 0.048 |
| *C. ultunense* | M_L_ < M_H_ | 4.43 | 0.047 |
| *S. schinkii* | M_L_ < M_H_ | 19.64 | <0.001 |
|  | Increase over time in M_H_/M_L_ | 24.11/25.24 | <0.001/<0.001 |
| *T. acetatoxydans* | M_L_ < M_H_ | 6.99 | 0.015 |
|  | Decrease over time in M_H_/ M_L2_ | 93.12/9.22 | <0.001/0.029 |
| pSAOB3 | M_L_ < M_H_ | 27.77 | <0.001 |
| pSAOB4 | M_L_ < M_H_ | 6.94 | 0.016 |
|  | Decrease over time in M_L_ | 13.68 | 0.003 |
| pSAOB5 | M_L_ < M_H_ | 4.46 | 0.046 |
|  | Decrease over time in M_L_ | 11.79 | 0.005 |
| pSAOB7 | M_L_ < M_H_ | 5.25 | 0.032 |
|  | Decrease over time in M_H_/M_L_ | 10.71/41.97 | 0.007/<0.001 |
| pSAOB9 | M_L_ < M_H_ | 9.73 | 0.010 |
| pSAOB10 | Decrease over time in M_H_/M_L_ | 11.58/49.54 | 0.005/<0.001 |
| **Diversity indices** |  |  |  |
| S_obs_ | Decrease over time in M_H_/M_L_ | 26.85/79.95 | <0.001/<0.001 |
| Chao | M_L_ < M_H_ | 6.30 | 0.016 |
|  | Decrease over time in M_H_/M_L_ | 31.59/39.60 | <0.001/<0.001 |
| Shannon | Decrease over time in M_H_/M_L_ | 25.99/78.92 | <0.001/<0.001 |
| **T-RFLP analysis** |  |  |  |
| 28 bp | Decrease over time in M_H1_ | 44.69 | <0.001 |
| 58 bp | Decrease over time in M_H2_ | 22.17 | <0.001 |
| 108 bp | Increase over time in M_H2_ | 12.63 | 0.003 |
| 216 bp | Decrease over time in M_H2_ | 44.28 | <0.001 |
| 328 bp | Decrease over time in M_H1_/M_H2_ | 18.00/11.98 | <0.001/0.003 |
| 406 bp | Increase over time in M_H1_/M_H2_ | 88.04/56.38 | 0.011/<0.001 |
| 472 bp | Increase over time in M_H2_ | 9.71 | 0.007 |
| 516 bp | Decrease over time in M_H2_ | 5.50 | 0.032 |

**Table S3.** Significant differences in quantitative PCR (qPCR) data and diversity indices between thermophilic (52°C) chemostats fed high (T_H_, 7.5 g L^-1^) and low (T_L_, 0.4 g L^-1^) acetate and over time. In chemostat comparisons, data from HRT 2-6 were included in analyses. Statistical analyses were conducted using the General Linear Model and significance was set to *P*<0.05

|  | | | **Difference** | | | | **F-value** | | **P** |
| --- | --- | --- | --- | --- | --- | --- | --- | --- | --- |
| Methanomicrobiales | | | T_L_ < T_H_ | | | | 24.28 | | <0.001 |
|  | | | Decrease over time in T_H_/T_L_ | | | | /9.80 | | /0.005 |
| Total bacteria (mL^-1^ sludge) | | | T_L_ > T_H_ | | | | 7.85 | | 0.008 |
|  | | | Decrease over time in T_H_/T_L2_ | | | | 15.55/19.8 | | <0.001/<0.001 |
| *T. phaeum* | | | Increase over time in T_L_ | | | | 25.30 | | <0.001 |
| *S. schinkii* | | | T_L_ < T_H_ | | | | 86.49 | | <0.001 |
|  | | | Decrease over time in T_L_ | | | | 51.62 | | <0.001 |
| *T. acetatoxydans* | | | T_L_ < T_H2_ | | | | 31.33 | | <0.001 |
| **Diversity indices** |  | | |  | |  | | |  |
| S_obs_ | | | Decrease over time in T_H_ | | | | 52.93 | | <0.001 |
| Chao | | | Decrease over time in T_H_ | | | | 44.78 | | <0.001 |
| Shannon | | | Decrease over time in T_H_ | | | | 47.83 | | <0.001 |
| **T-RFLP analysis** | |  | | |  | | |  | |
| 60 bp | | | Increase over time in T_H2_ | | | | 8.60 | | 0.010 |
| 80 bp | | | Decrease over time in T_H1_ | | | | 6.45 | | 0.022 |
|  |  |  | Increase over time in T_H2_ | | | | 80.62 | | <0.001 |
| 168 bp | | | Decrease over time in T_H2_ | | | | 14.18 | | 0.002 |
| 264 bp | | | Decrease over time in T_H2_ | | | | 25.54 | | <0.001 |
| 268 bp | | | Increase over time in T_H2_ | | | | 9.71 | | 0.008 |
| 300 bp | | | Decrease over time in T_H1_/T_H2_ | | | | 11.26/27.74 | | 0.004/<0.001 |
| 304 bp | | | Decrease over time in T_H1_/T_H2_ | | | | 57.67/60.49 | | <0.001/<0.001 |
| 484 bp | | | Increase over time in T_H1_ | | | | 4.99 | | 0.040 |
| 536 bp | | | Increase over time in T_H1_/T_H2_ | | | | 42.13/5.94 | | <0.001/0.030 |
| 632 bp | | | Decrease over time in T_H1_ | | | | 10.03 | | 0.006 |
| 636 bp | | | Increase over time in T_H1_ | | | | 20.43 | | <0.001 |

**Table S4.** Significant differences in acetate levels, richness and evenness indices between mesophilic (M) and thermophilic (T) high-acetate (_H_, 7.5 g L^-1^) chemostats. In comparison of acetate levels, data from HRT 2-6 were included in the analyses. Statistical analyses were conducted using the General Linear Model and significance was set to *P*<0.05

|  | **Difference** | **F-value** | **P** |
| --- | --- | --- | --- |
| Acetate concentration | T_H_ > M_H_ | 15.96 | <0.001 |
| **Diversity indices** | | | |
| S_obs_ HRT 1 | M_H_ > T_H_ | 45.66 | <0.001 |
| S_obs_ HRT 6 | M_H_ > T_H_ | 50.15 | <0.001 |
| Chao HRT 1 | M_H_ > T_H_ | 14.72 | 0.001 |
| Chao HRT 6 | M_H_ > T_H_ | 23.27 | <0.001 |
| Shannon HRT 1 | M_H_ > T_H_ | 39.81 | <0.001 |

**Table S5.** Accession numbers of *fhs* sequences retrived in clone libraries from the chemostats M_H1_ M_H2_, T_H1_ and T_H2_ after 6 hydraulic retention times of operation (HRT 28 days).

| **Accession number(s)** | **Assignment in T-RFLP and phylogenetic tree**  **[in clone library]^1^** | **Chemostats**  **(number of clones)** | **Close relatedness to previous sequences (accession number)** |
| --- | --- | --- | --- |
|  |  |  |  |
| **Mesophilic high-acetate chemostats M_H_** | | | |
| MG012806-19 | 28bp [OTU M_H_ 28] | M_H1_ (5), M_H2_ (9) | Uncult bacterium SAO3_cl5, cl41, cl51 (99%, JQ082262/93/67)^2^  Uncult bacterium N5_c59 (99%, KP184585)^3^  Uncult bacterium OTU5_cl6,8, cl33, cl70 (99%, KJ701094/95/98/97)^4^ |
| MG012823 | 58bp [OTU M_H_ 58_1] | M_H1_ (1) | Uncult bacterium SAO3_cl6, cl27 (81-82%, JQ082257/94)^2^ |
| MG012820-22 | 58bp [OTU M_H_ 58_2] | M_H1_ (2), M_H2_ (1) | Uncult bacterium SAO3_cl18 (97%, JQ082259)^1^, Uncult bacterium clN5_c43 (97%, KP184577)^3^  Aminobacterium colombiense (97%, CP001997) |
| MG012824 | 99bp [OTU M_H_ 99bp] | M_H1_ (1) | *Clostridium ultunense* strain Esp fhs1 (81%, JQ979075)  Uncult bacterium SAO3_cl35 (80%, JQ082286)^2^ |
| MG012825 | 108bp [OTU M_H_ 108_2] | M_H2_ (1) | Uncult bacterium SAO3_cl6, cl27 (81%, JQ082257/94)^2^ |
| MG012826 | na [OTU M_H_ 134bp] | M_H1_ (1) | Uncult bacterium SAO3_cl28 (99%, JQ082275)^2^ |
| MG012827 | 328bp [OTU M_H_ 328] | M_H1_ (1) | Uncult bacterium O1_c59 (98%, KP184566)^3^  Uncult bacterium cl31 (98%, KJ701075)^4^ |
| MG012828-40 | 406 bp [OTU M_H_ 406] | M_H1_ (6) M_H2_ (7) | <79% |
| **Thermophilic high-acetate chemostats T_H_** | | | |
| MG385064-70 | 60bp [OTU T_H_ 60] | T_H1_ (4) T_H2_ (3) | <79% |
| MG385071-73 | 80bp [OTU T_H_ 80] | T_H2_ (3) | Uncult bacterium SAO3_cl30 (97%, JQ082288)^2^  *Tepidanaerobacter acetatoxydans* Re1 fhs1 (96%, HF563609) |
| MG385074 | 132bp [OTU T_H_ 132bp] | T_H1_ (1) | <79% |

^1^na = no assignment in T-RFLP.

^2^(Müller et al., 2016).

^3^(Moestedt et al., 2016).

^4^(Westerholm et al., 2015).

**Table S6.**

Operating conditions for the two biogas digesters that were the inoculum source for the mesophilic (37°C) and thermophilic (52°C) chemostats in the present study

| **Trial** | **Mesophilic** | **Thermophilic** |
| --- | --- | --- |
| Digester affiliation | D^TE^37^a^ | Kungsängens gård |
| Substrate | Source-sorted organic fraction of municipal solid waste | Industrial food waste, manure |
| Temperature (°C) | 37 | 52 |
| Organic loading rate (OLR, gVS/day/L) | 2.3 | 3.5 |
| Hydraulic retention time (HRT, days) | 30 | 3 |
| NH_4_^+^-N (g/L) | 5.4 | 3.2 |
| NH_3_ (g/L) | 0.6-0.9 | 0.6 |
| Supplementation | Egg albumin | - |
| Trace element supplementation | 0.009 L Kemira BDP-866/kg digester sludge | 1.5 L Kemira BDP-866/m^3^ substrate |
| Volatile fatty acid (VFA) concentration | >0.1 | 3.8 |

^a^For further description, see Westerholm et al. (2015).

**Table S7.** Sample name in Illumina sequencing data, sequencing results, source chemostat and the hydraulic retention time (HRT) for sampling.

| **Sample name in MiSeq data** | **Raw read pairs** | **High quality read pairs** | **Chemostat** | **HRT** |
| --- | --- | --- | --- | --- |
| S1 | 86 433 | 25 145 | T_H1_ |  |
| S2 | 103 408 | 30 111 |  | 1 |
| S3 | 99 280 | 31 499 |  |  |
| S4 | 82 596 | 26 477 | T_H1_ |  |
| S5 | 94 591 | 25 323 |  | 2 |
| S6 | 102 770 | 30 164 |  |  |
| S7 | 90 752 | 27 888 | T_H1_ |  |
| S8 | 83 482 | 25 116 |  | 3 |
| S9 | 78 647 | 22 293 |  |  |
| S10 | 95 369 | 27 500 | T_H1_ |  |
| S11 | 74 453 | 22 161 |  | 3 |
| S12 | 61 086 | 14 474 |  |  |
| S13 | 88 860 | 27 771 | T_H1_ |  |
| S14 | 61 885 | 21 617 |  | 4 |
| S15 | 81 088 | 23 311 |  |  |
| S16 | 103 817 | 33 546 | T_H1_ |  |
| S17 | 102 515 | 31 836 |  | 4 |
| S18 | 74 868 | 22 789 |  |  |
| S19 | 76 597 | 21 595 | T_H1_ |  |
| S20 | 82 886 | 23 159 |  | 4 |
| S21 | 87 697 | 28 735 |  |  |
| S22 | 103 765 | 31 434 | T_H1_ | 5 |
| S23 | 85 689 | 24 430 |  |  |
| S24 | 71 670 | 26 138 |  |  |
| S25 | 75 563 | 23 322 | T_H1_ |  |
| S26 | 89 349 | 30 524 |  | 6 |
| S27 | 74 026 | 24 544 |  |  |
| S28 | 31 801 | 10 608 | T_H1_ |  |
| S29 | 56 276 | 17 761 |  | 6 |
| S30 | 57 470 | 18 132 |  |  |
| S31 | 59 969 | 18 927 | T_H1_ |  |
| S32 | 82 775 | 24 472 |  | 6 |
| S33 | 64 895 | 20 878 |  |  |
| S34 | 81 520 | 25 045 | T_H2_ | 1 |
| S35 | 4 258 | 1 082 |  |  |
| S36 | 93 705 | 28 612 | T_H2_ |  |
| S37 | 91 671 | 25 831 |  | 1 |
| S38 | 72 027 | 19 500 |  |  |
| S39 | 75 115 | 19 599 | T_H2_ |  |
| S40 | 74 524 | 18 698 |  | 1 |
| S41 | 61 588 | 18 579 |  |  |
| S42 | 106 552 | 29 503 | T_H2_ |  |
| S43 | 78 741 | 23 771 |  | 2 |
| S44 | 68 059 | 22 922 |  |  |
| S45 | 82 231 | 21 810 | T_H2_ |  |
| S46 | 75 922 | 24 865 |  | 3 |
| S47 | 69 508 | 21 779 |  |  |
| S48 | 61 227 | 18 711 | T_H2_ |  |
| S49 | 65 742 | 19 836 |  | 3 |
| S50 | 65 761 | 19 744 |  |  |
| S51 | 46 054 | 14 189 | T_H2_ |  |
| S52 | 70 938 | 19 857 |  | 4 |
| S53 | 57 599 | 17 641 |  |  |
| S54 | 46 823 | 15 800 | T_H2_ |  |
| S55 | 68 553 | 19 828 |  | 4 |
| S56 | 53 511 | 15 814 |  |  |
| S57 | 56 648 | 17 423 | T_H2_ |  |
| S58 | 58 093 | 16 837 |  | 4 |
| S59 | 62 416 | 17 163 |  |  |
| S60 | 74 936 | 20 832 | T_H2_ |  |
| S61 | 75 391 | 23 583 |  | 5 |
| S62 | 73 484 | 21 075 |  |  |
| S63 | 77 067 | 23 619 | T_H2_ |  |
| S64 | 66 097 | 24 154 |  | 6 |
| S65 | 86 577 | 26 459 |  |  |
| S66 | 80 828 | 27 736 | T_H2_ |  |
| S67 | 82 011 | 24 436 |  | 6 |
| S68 | 72 655 | 23 639 |  |  |
| S69 | 49 043 | 14 950 | T_H2_ |  |
| S70 | 68 853 | 20 644 |  | 6 |
| S71 | 70 025 | 24 735 |  |  |
| S72 | 86 138 | 27 792 | T_L1_ |  |
| S73 | 71 013 | 21 514 |  | 1 |
| S74 | 64 579 | 21 775 |  |  |
| S75 | 77 988 | 21 960 | T_L1_ |  |
| S76 | 67 392 | 20 335 |  | 1 |
| S77 | 73 217 | 21 239 |  |  |
| S78 | 57 072 | 14 446 | T_L1_ | 1 |
| S79 | 61 279 | 15 689 |  |  |
| S80 | 65 443 | 16 393 |  |  |
| S81 | 54 193 | 14 693 | T_L1_ |  |
| S82 | 58 358 | 14 120 |  | 2 |
| S83 | 59 839 | 15 916 |  |  |
| S84 | 49 962 | 14 878 | T_L1_ |  |
| S85 | 47 062 | 12 240 |  | 3 |
| S86 | 53 795 | 15 591 |  |  |
| S87 | 51 834 | 14 459 | T_L1_ | 3 |
| S88 | 64 779 | 10 105 |  |  |
| S89 | 55 907 | 6 865 | T_L1_ | 4 |
| S90 | 66 054 | 9 378 |  |  |
| S91 | 53 587 | 9 210 |  |  |
| S92 | 56 986 | 8 420 | T_L1_ |  |
| S93 | 44 470 | 7 473 |  | 4 |
| S94 | 58 864 | 11 124 |  |  |
| S95 | 75 719 | 11 616 | T_L1_ |  |
| S96 | 57 812 | 9 853 |  | 4 |
| S97 | 67 987 | 10 674 |  |  |
| S98 | 71 153 | 10 816 | T_L1_ | 5 |
| S99 | 51 658 | 7 168 |  |  |
| S100 | 53 000 | 5 772 | T_L1_ | 6 |
| S102 | 56 726 | 7 090 |  |  |
| S103 | 76 408 | 10 316 | T_L1_ |  |
| S104 | 61 424 | 10 271 |  | 6 |
| S105 | 83 788 | 11 093 |  |  |
| S106 | 72 801 | 12 164 | T_L1_ | 6 |
| S107 | 83 418 | 12 684 |  |  |
| S108 | 79 764 | 11 363 |  |  |
| S109 | 49 244 | 7 131 | T_L2_ |  |
| S110 | 69 813 | 10 039 |  | 1 |
| S111 | 65 589 | 10 785 |  |  |
| S112 | 77 543 | 20 335 | T_L2_ | 1 |
| S113 | 65 432 | 18 797 |  |  |
| S114 | 63 912 | 20 084 |  |  |
| S115 | 97 290 | 24 640 | T_L2_ |  |
| S116 | 91 538 | 26 130 |  | 1 |
| S117 | 72 700 | 19 923 |  |  |
| S118 | 58 372 | 14 766 | T_L2_ |  |
| S119 | 51 235 | 12 189 |  | 2 |
| S120 | 58 540 | 13 780 |  |  |
| S121 | 61 510 | 17 967 | T_L2_ |  |
| S122 | 72 643 | 19 161 |  | 3 |
| S123 | 70 698 | 20 581 |  |  |
| S124 | 57 267 | 19 272 | T_L2_ |  |
| S125 | 64 640 | 18 459 |  | 3 |
| S126 | 73 568 | 23 413 |  |  |
| S127 | 84 402 | 24 806 | T_L2_ |  |
| S128 | 75 900 | 21 834 |  | 4 |
| S129 | 54 504 | 14 766 |  |  |
| S130 | 66 343 | 16 650 | T_L2_ |  |
| S131 | 68 697 | 17 537 |  | 4 |
| S132 | 81 269 | 18 012 |  |  |
| S133 | 83 163 | 20 550 | T_L2_ |  |
| S134 | 65 227 | 18 498 |  | 4 |
| S135 | 85 840 | 19 654 |  |  |
| S136 | 71 585 | 18 623 | T_L2_ | 5 |
| S137 | 75 413 | 18 436 |  |  |
| S138 | 68 071 | 16 657 |  |  |
| S139 | 65 545 | 14 556 | T_L2_ | 6 |
| S140 | 85 338 | 19 234 |  |  |
| S141 | 66 327 | 18 878 |  |  |
| S142 | 75 538 | 19 968 | T_L2_ |  |
| S143 | 72 251 | 21 052 |  | 6 |
| S144 | 66 784 | 22 092 |  |  |
| S145 | 76 964 | 20 558 | T_L2_ |  |
| S146 | 69 596 | 21 028 |  | 6 |
| S147 | 90 311 | 25 859 |  |  |
| S148 | 76 523 | 21 900 | M_H1_ |  |
| S149 | 71 272 | 18 881 |  | 1 |
| S150 | 97 447 | 25 879 |  |  |
| S151 | 73 897 | 21 507 | M_H1_ |  |
| S152 | 55 814 | 14 731 |  | 1 |
| S153 | 72 576 | 20 506 |  |  |
| S154 | 56 766 | 18 255 | M_H1_ |  |
| S155 | 58 252 | 15 195 |  | 2 |
| S156 | 58 943 | 17 719 |  |  |
| S157 | 54 080 | 15 252 | M_H1_ |  |
| S158 | 83 324 | 22 585 |  | 3 |
| S159 | 63 949 | 16 527 |  |  |
| S160 | 58 388 | 15 498 | M_H1_ |  |
| S161 | 66 890 | 19 176 |  | 4 |
| S162 | 100 868 | 25 247 |  |  |
| S163 | 68 741 | 18 413 | M_H1_ | 5 |
| S164 | 75 891 | 23 236 |  |  |
| S165 | 83 448 | 20 121 |  |  |
| S166 | 65 262 | 18 504 | M_H1_ |  |
| S167 | 60 863 | 16 217 |  | 6 |
| S168 | 88 290 | 23 004 |  |  |
| S169 | 73 765 | 18 572 | M_H2_ |  |
| S170 | 78 959 | 19 213 |  | 1 |
| S171 | 74 266 | 24 550 |  |  |
| S172 | 89 344 | 27 073 | M_H2_ |  |
| S173 | 76 635 | 24 513 |  | 2 |
| S174 | 56 661 | 20 475 |  |  |
| S175 | 64 162 | 19 260 | M_H2_ |  |
| S176 | 100 424 | 34 584 |  | 2 |
| S177 | 36 597 | 12 046 |  |  |
| S178 | 96 266 | 31 909 | M_H2_ | 3 |
| S179 | 81 501 | 25 266 |  |  |
| S180 | 84 307 | 26 032 |  |  |
| S181 | 72 312 | 21 388 | M_H2_ |  |
| S182 | 74 001 | 18 868 |  | 4 |
| S183 | 103 762 | 29 542 |  |  |
| S184 | 64 883 | 20 096 | M_H2_ | 3 |
| S185 | 84 738 | 20 628 |  |  |
| S186 | 79 967 | 23 367 | M_H2_ | 3 |
| S187 | 68 909 | 18 620 |  |  |
| S188 | 79 697 | 20 896 | M_L1_ |  |
| S191 | 67 588 | 20 059 |  | 1 |
| S192 | 82 380 | 21 991 |  |  |
| S193 | 87 661 | 25 731 | M_L1_ | 1 |
| S194 | 76 914 | 25 316 |  |  |
| S195 | 98 057 | 26 261 |  |  |
| S196 | 89 757 | 27 016 | M_L1_ |  |
| S197 | 118 785 | 33 382 |  | 2 |
| S198 | 17 734 | 4 873 |  |  |
| S199 | 89 638 | 22 962 | M_L1_ |  |
| S200 | 64 952 | 16 712 |  | 3 |
| S201 | 93 498 | 28 282 |  |  |
| S202 | 92 589 | 23 086 | M_L1_ |  |
| S203 | 121 675 | 37 859 |  | 4 |
| S204 | 84 857 | 25 315 |  |  |
| S205 | 83 744 | 24 937 | M_L1_ |  |
| S206 | 80 166 | 28 030 |  | 5 |
| S207 | 87 786 | 28 204 |  |  |
| S208 | 91 518 | 31 729 | M_L1_ | 6 |
| S209 | 98 249 | 32 676 |  |  |
| S210 | 94 110 | 24 513 |  |  |
| S211 | 70 855 | 22 376 | M_L2_ |  |
| S212 | 84 729 | 25 638 |  | 1 |
| S213 | 75 401 | 22 078 |  |  |
| S214 | 83 097 | 24 692 | M_L2_ |  |
| S215 | 110 109 | 29 658 |  | 2 |
| S216 | 106 681 | 29 833 |  |  |
| S217 | 99 033 | 31 176 | M_L2_ |  |
| S218 | 75 613 | 18 666 |  | 2 |
| S219 | 84 785 | 26 222 |  |  |
| S220 | 60 419 | 18 260 | M_L2_ | 3 |
| S221 | 79 828 | 22 724 |  |  |
| S222 | 82 136 | 27 606 |  |  |
| S223 | 98 345 | 32 780 | M_L2_ |  |
| S224 | 83 171 | 28 552 |  | 4 |
| S225 | 102 124 | 33 323 |  |  |
| S226 | 74 481 | 19 140 | M_L2_ |  |
| S227 | 82 978 | 26 888 |  | 5 |
| S228 | 97 738 | 29 927 |  |  |
| S229 | 94 562 | 28 599 | M_L2_ |  |
| S230 | 96 887 | 34 282 |  | 6 |
| S231 | 102 175 | 33 407 |  |  |

**References**

Moestedt, J., Müller, B., Westerholm, M., Schnürer, A. (2016) Ammonia threshold for inhibition of anaerobic digestion of thin stillage and the importance of organic loading rate. *Microbial Biotechnol* **9**: 180-194.

Müller, B., Sun, L., Westerholm, M., Schnürer, A. (2016) Bacterial community composition and *fhs* profiles of low- and high-ammonia biogas digesters reveal novel syntrophic acetate-oxidising bacteria. *Biotechnol Biofuel* **9:** 1-18.

Westerholm, M., Müller, B., Isaksson, S., Schnürer, A., 2015. Trace element and temperature effects on microbial communities and links to biogas digester performance at high ammonia levels. *Biotechnol Biofuel* **8**: 1-19.

**Appendix 1**

**Scripts used for processing sequence reads in 16S rRNA analysis**

**Cutadapt pre-processing of sequence reads**

#!/bin/bash

#Cutadapt script for celaning 16S data, requires already demplutiplexed data in the form of SAMPLE_R*_001.fastq.gz. Designed for paired end.

exec 3>&1 4>&2

trap 'exec 2>&4 1>&3' 0 1 2 3

exec 1>cutadapt.out 2>&1

mkdir -p OUTPUT_DIRECTORY #Name your output directory here

for file in *_R1_001.fastq.gz; do

prefix=${file%_R1_001.fastq.gz};

FwdIn=${prefix}_R1_001.fastq.gz;

RevIn=${prefix}_R2_001.fastq.gz;

FwdOut=${prefix}_trimmed_R1_001.fastq.gz

RevOut=${prefix}_trimmed_R2_001.fastq.gz

cutadapt -g GTGBCAGCMGCCGCGGTAA -G GACTACHVGGGTATCTAATCC --max-n 0 --maximum-length 300 --minimum-length 250 --pair-filter=both --discard-untrimmed -q 30 -o ./trimmed/${FwdOut} -p ./trimmed/${RevOut} ${FwdIn} ${RevIn};

done;

**Sickle -pre-processing of sequence reads**

#!/bin/bash

#Sickle script for cleaning 16S data, requires already demplutiplexed data in the form of SAMPLE_R*_001.fastq.gz. Designed for paired end. Data is previously processed with cutadapt.

exec 3>&1 4>&2

trap 'exec 2>&4 1>&3' 0 1 2 3

exec 1>sickle.out 2>&1

mkdir -p OUTPUT_DIRECTORY #Name your output directory here

cd Analysis

path_out=$PWD

cd ".."

#cd trimmed

for file in *_R1_001.fastq.gz; do

prefix=${file%_R1_001.fastq.gz};

FwdIn=${prefix}_R1_001.fastq.gz;

RevIn=${prefix}_R2_001.fastq.gz;

FwdOut=${prefix}_filterd_R1_001.fastq.gz

RevOut=${prefix}_filterd_R2_001.fastq.gz

Single=${prefix}_singles.fastq.gz

sickle pe -f ${FwdIn} -r ${RevIn} -q 30 -t sanger -o ${path_out}/${FwdOut} -p ${path_out}/${RevOut} -s ${path_out}/${Single} -l 180 -t sanger

done;

**Mothur batch mode processing of reads according to MiSeq SOP**

#Begin Batch file, preprocessing according to MiSeq SOP

set.dir(output=./)

make.file(inputdir=/path/to/data/, type=gz, prefix=Analysis)

set.dir(input=./)

make.contigs(file=Analysis.files, processors=48)

summary.seqs(fasta=/path/to/data/Analysis.trim.contigs.fasta)

screen.seqs(fasta=/path/to/data/Analysis.trim.contigs.fasta, group=/path/to/data/Analysis.contigs.groups, maxambig=0, maxlength=300)

unique.seqs(fasta=/path/to/data/Analysis.trim.contigs.good.fasta)

count.seqs(name=/path/to/data/Analysis.trim.contigs.good.names, group=/path/to/data/Analysis.contigs.good.groups)

summary.seqs(count=/path/to/data/Analysis.trim.contigs.good.count_table)

pcr.seqs(fasta=/path/to/data/silva.nr_v128.align, oligos=/path/to/data/Enrich.oligos, keepdots=F)

system(mv /path/to/data/silva.nr_v128.pcr.align /path/to/data/silva.custom.fasta)

align.seqs(fasta=Analysis.trim.contigs.good.unique.fasta, reference=silva.custom.fasta)

summary.seqs(fasta=/path/to/data/Analysis.trim.contigs.good.unique.align, count=/path/to/data/Analysis.trim.contigs.good.count_table)

screen.seqs(fasta=/path/to/data/Analysis.trim.contigs.good.unique.align, count=/path/to/data/Analysis.trim.contigs.good.count_table, summary=/path/to/data/Analysis.trim.contigs.good.unique.summary, start=12749, end=22327, maxhomop=8)

summary.seqs(fasta=current, count=current)

filter.seqs(fasta=/path/to/data/Analysis.trim.contigs.good.unique.good.align, vertical=T, trump=.)

unique.seqs(fasta=/path/to/data/Analysis.trim.contigs.good.unique.good.filter.fasta, count=/path/to/data/Analysis.trim.contigs.good.good.count_table)

pre.cluster(fasta=/path/to/data/Analysis.trim.contigs.good.unique.good.filter.unique.fasta, count=/path/to/data/Analysis.trim.contigs.good.unique.good.filter.count_table, diffs=2)

chimera.vsearch(fasta=/path/to/data/Analysis.trim.contigs.good.unique.good.filter.unique.precluster.fasta, count=/path/to/data/Analysis.trim.contigs.good.unique.good.filter.unique.precluster.count_table, dereplicate=t)

remove.seqs(fasta=/path/to/data/Analysis.trim.contigs.good.unique.good.filter.unique.precluster.fasta, accnos=/path/to/data/Analysis.trim.contigs.good.unique.good.filter.unique.precluster.denovo.vsearch.accnos, name=/path/to/data/Analysis.trim.contigs.good.names)

summary.seqs(fasta=current, count=current)

classify.seqs(fasta=/path/to/data/Analysis.trim.contigs.good.unique.good.filter.unique.precluster.pick.fasta, count=/path/to/data/Analysis.trim.contigs.good.unique.good.filter.unique.precluster.denovo.vsearch.pick.count_table, reference=/path/to/data/trainset16_022016.rdp.fasta, taxonomy=/path/to/data/trainset16_022016.rdp.tax, cutoff=80)

remove.lineage(fasta=/path/to/data/Analysis.trim.contigs.good.unique.good.filter.unique.precluster.pick.fasta, count=/path/to/data/Analysis.trim.contigs.good.unique.good.filter.unique.precluster.denovo.vsearch.pick.count_table, taxonomy=/path/to/data/Analysis.trim.contigs.good.unique.good.filter.unique.precluster.pick.rdp.wang.taxonomy, taxon=Chloroplast-Mitochondria-unknown-Eukaryota)

summary.tax(taxonomy=current, count=current)

dist.seqs(fasta=current, cutoff=0.03, processors=48)

cluster.split(fasta=current, count=current, taxonomy=current, splitmethod=classify, taxlevel=4, cutoff=0.03)

make.shared(list=current, count=current, label=0.03)

classify.otu(list=current, count=current, taxonomy=current, label=0.03)

count.groups(shared=current)
